# Supplementary material for: Epigenome-wide association study of serum urate reveals insights into urate co-regulation and the SLC2A9 locus
Source: Nat Commun. 2021 Dec 9;12:7173. doi: 10.1038/s41467-021-27198-4 (PMC8660809; doi:10.1038/s41467-021-27198-4)

Epigenome-wide association study of serum urate reveals insights into urate co-regulation and the *SLC2A9* locus

Supplementary Information

Supplementary Note 1: Cohort descriptions and acknowledgments

ARIC

The Atherosclerosis Risk in Communities study has been funded in whole or in part with Federal funds from the National Heart, Lung, and Blood Institute, National Institutes of Health, Department of Health and Human Services (contract numbers HHSN268201700001I, HHSN268201700002I, HHSN268201700003I, HHSN268201700004I and HHSN268201700005I), R01HL087641, R01HL059367 and R01HL086694; National Human Genome Research Institute contract U01HG004402; and National Institutes of Health contract HHSN268200625226C. Funding was also supported by 5RC2HL102419, R01NS087541 and R01HL131136. The authors thank the staff and participants of the ARIC study for their important contributions. Infrastructure was partly supported by Grant Number UL1RR025005, a component of the National Institutes of Health and NIH Roadmap for Medical Research. The work of Anna Köttgen was funded by the Deutsche Forschungsgemeinschaft (DFG, German Research Foundation) Project-ID 431984000 – SFB 1453 and Project-ID 192904750 – SFB 992. Adrienne Tin is supported by NIAMS grant R01AR073178-01A1. The work of Pascal Schlosser was funded by DFG Project-ID 192904750 – SFB 992 and the EQUIP Program for Medical Scientists, Faculty of Medicine, University of Freiburg.

CARDIA

The CARDIA study is a prospective multicenter study with 5,115 adult Caucasian and African-American participants ages 18–30 years at recruitment. The recruitment was done from four centers as follows: the total community in Birmingham, AL; selected census tracts in Chicago, IL, and Minneapolis, MN; and from the Kaiser Permanente health plan membership in Oakland, CA. Details of the CARDIA study design have been previously published (1, 2). Nighttime examinations were completed beginning with the study initiation in 1985–1986 and in follow-up years 0, 2, 5, 7, 10, 15, 20, 25, and 30.

The Coronary Artery Risk Development in Young Adults Study (CARDIA) is supported by contracts HHSN268201800003I, HHSN268201800004I, HHSN268201800005I, HHSN268201800006I, and HHSN268201800007I from the National Heart, Lung, and Blood Institute (NHLBI).

CHS

This CHS research was supported by NHLBI contracts HHSN268201200036C, HHSN268200800007C, HHSN268201800001C, N01HC55222, N01HC85079, N01HC85080, N01HC85081, N01HC85082, N01HC85083, N01HC85086, 75N92021D00006; and NHLBI grants U01HL080295, R01HL087652, R01HL105756, R01HL103612, R01HL120393, R01HL085251, and U01HL130114 with additional contribution from the National Institute of Neurological Disorders and Stroke (NINDS). Additional support was provided through R01AG023629 from the National Institute on Aging (NIA). A full list of principal CHS investigators and institutions can be found at CHS-NHLBI.org.

The provision of genotyping data was supported in part by the National Center for Advancing Translational Sciences, CTSI grant UL1TR001881, and the National Institute of Diabetes and Digestive and Kidney Disease Diabetes Research Center (DRC) grant DK063491 to the Southern California Diabetes Endocrinology Research Center.

The content is solely the responsibility of the authors and does not necessarily represent the official views of the National Institutes of Health.

EstBB

The Estonian Biobank is a population-based biobank of the Estonian Genome Center at the University of Tartu (EstBB). The cohort size is currently close to 200,000 participants aged ≥18, which closely reflects the age, sex and geographical distribution of the Estonian population. The samples used in this study were selected from the EstBB Center for Translational Genomics (CTG) cohort of individuals who have been recontacted for a second time-point sample (EstBB-CTG), and an aging study of 50 younger (age 22–34) and 50 older (age 73–84) individuals (EstBB-YO). The research of the EstBB cohort was supported by the European Union through the European Regional Development Fund (Project No. 2014-2020.4.01.15-0012). Data analyses was carried out in part in the High-Performance Computing Center of University of Tartu.

Estonian Biobank Research Team: group author acknowledgement

Tõnu Esko, Andres Metspalu, Reedik Mägi, Mari Nelis

ESTHER

ESTHER is a statewide population-based cohort study conducted in Saarland/Germany. In 2000-2002, 9,940 older adults (50-75 years) were recruited and have been regularly followed up since then. Recruitment and collection of baseline data used for this study have been supported by a grant from the Baden-Württemberg state Ministry of Science, Research and Arts. This study included a random sample of ESTHER participants (n=485) for whom epigenome wide DNA profiling was performed.

The ESTHER study was supported by the Baden-Württemberg State Ministry of Science, Research and Arts (Stuttgart, Germany), the Federal Ministry of Education and Research (Berlin, Germany) and the Federal Ministry of Family Affairs, Senior Citizens, Women and Youth (Berlin, Germany). The participants completed a standardized self-administered questionnaire and donated biological samples (blood, stool and urine) during baseline enrolment. Comprehensive medical data, such as the results of a physical assessment, medical diagnoses and drug prescriptions were additionally obtained from the general practitioner. The ESTHER study was approved by the Ethics Committees of the Medical Faculty of the University of Heidelberg and of the Physicians' Board of Saarland.

GENOA

The Genetic Epidemiology Network of Arteriopathy (GENOA) study is a community-based study of hypertensive sibships that was designed to investigate the genetics of hypertension and target organ damage in African Americans from Jackson, Mississippi and non-Hispanic whites from Rochester, Minnesota (Daniels, 2004). In the initial phase of the GENOA study (Phase I: 1996-2001), all members of sibships containing ≥ 2 individuals with essential hypertension clinically diagnosed before age 60 were invited to participate, including both hypertensive and normotensive siblings. Exclusion criteria of the GENOA study were secondary hypertension, alcoholism or drug abuse, pregnancy, insulin-dependent diabetes mellitus, or active malignancy. Eighty percent of African Americans (1,482 subjects) and 75% of non-Hispanic whites (1,213 subjects) from the initial study population returned for the second examination (Phase II: 2001-2005). Study visits were made in the morning after an overnight fast of at least eight hours. Demographic information, medical history, clinical characteristics, lifestyle factors, and blood samples were collected in each phase. Written informed consent was obtained from all subjects and approval was granted by participating institutional review boards. DNA methylation levels were measured only in African Americans participants, so participants in the current analysis are African American. Participants were excluded from this analysis if they were also participants in the Atherosclerosis Risk in Communities (ARIC) study. Methylation and all kidney-related measures were from the Phase II examination. Support for GENOA was provided by the National Heart, Lung and Blood Institute (U01 HL054457, RC1 HL100185, R01 HL119443, and R01 HL133221) and the National Institute of Diabetes and Digestive and Kidney Diseases (R01 DK073537) of the National Institutes of Health. We appreciate technical assistance from Stephen T. Turner, Pamela I. Hammond, Julie M. Cunningham, and the Mayo Clinic Advanced Genomics Technology Center. We would also like to thank the families that participated in the GENOA study.

JHS

The JHS is a large, population-based observational study evaluating the etiology of cardiovascular, renal, and respiratory diseases among African Americans residing in the three counties (Hinds, Madison, and Rankin) that make up the Jackson, Mississippi metropolitan area. Data and biologic materials have been collected from 5306 participants, including a nested family cohort of 1,498 members of 264 families. The age at enrollment for the unrelated cohort was 35-84 years; the family cohort included related individuals >21 years old. Participants provided extensive medical and social history, had an array of physical and biochemical measurements and diagnostic procedures, and provided genomic DNA during a baseline examination (2000-2004) and two follow-up examinations (2005-2008 and 2009-2012). The study population is characterized by a high prevalence of diabetes, hypertension, obesity, and related disorders. Annual follow-up interviews and cohort surveillance are ongoing.

**KORA F4**	The KORA research platform (KORA, Cooperative Health Research in the Region of Augsburg) was initiated and financed by the Helmholtz Zentrum München - German Research Center for Environmental Health, which is funded by the German Federal Ministry of Education and Research and by the State of Bavaria. Furthermore, KORA research was supported within the Munich Center of Health Sciences (MC Health), Ludwig-Maximilians-Universität, as part of LMUinnovativ. The German Diabetes Center is supported by the Ministry of Culture and Science of the state of North Rhine-Westphalia (Düsseldorf, Germany) and the German Federal Ministry of Health (Berlin, Germany). This study was supported in part by a grant from the German Federal Ministry of Education and Research to the German Center for Diabetes Research (DZD).
**Lothian Birth Cohort, 1936**	The Lothian Birth Cohort (LBC) 1936 study is designed as a follow-up cohort study. The participants comprise surviving members of the Scottish Mental Survey of 1947 (SMS1947; N = 70,805) who reside in the Edinburgh area (Lothian) of Scotland. The SMS1947 applied a valid test of general intelligence to all children born in 1936 and attending Scottish schools in June 1947. A total of 1091 participants make up the LBC 1936. They undertook: a medical interview and examination; physical fitness testing; extensive cognitive testing (reasoning, memory, speed of information processing, and executive function); personality, quality of life and other psycho-social questionnaires; and a food frequency questionnaire. They have taken the same mental ability test (the Moray House Test No. 12) at age 11 and age 70. They provided blood samples for DNA extraction and testing and other biomarker analyses. They have been followed up triennially to age 82; data for the current manuscript were taken from the second wave of the study (N = 866 at mean age ~73 years). The present analysis included participants with data in all variables (n=290). The LBC1936 is supported by Age UK (Disconnected Mind project, which supports S.E.H.), the Medical Research Council (G0701120, G1001245, MR/M013111/1, MR/R024065/1, which supports S.R.C.), and the University of Edinburgh. Methylation typing of LBC1936 was supported by Centre for Cognitive Ageing and Cognitive Epidemiology (Pilot Fund award), Age UK, The Wellcome Trust Institutional Strategic Support Fund, The University of Edinburgh, and The University of Queensland. We thank the Lothian Birth Cohort 1936 members who took part in this study, and Lothian Birth Cohort 1936 research team members who collected, entered and checked data used in this paper.

LOLIPOP

The LOLIPOP study is supported by the National Institute for Health Research (NIHR) Comprehensive Biomedical Research Centre Imperial College Healthcare NHS Trust, the British Heart Foundation (SP/04/002), the Medical Research Council (G0601966, G0700931), the Wellcome Trust (084723/Z/08/Z, 090532 & 098381) the NIHR (RP-PG-0407-10371), the NIHR Official Development Assistance (ODA, award 16/136/68), the European Union FP7 (EpiMigrant, 279143) and H2020 programs (iHealth-T2D, 643774). We acknowledge support of the MRC-PHE Centre for Environment and Health, and the NIHR Health Protection Research Unit on Health Impact of Environmental Hazards. The work was carried out in part at the NIHR/Wellcome Trust Imperial Clinical Research Facility. The views expressed are those of the author(s) and not necessarily those of the Imperial College Healthcare NHS Trust, the NHS, the NIHR or the Department of Health. We thank the participants and research staff who made the study possible. JC is supported by the Singapore Ministry of Health's National Medical Research Council under its Singapore Translational Research Investigator (STaR) Award (NMRC/STaR/0028/2017).

LURIC

LURIC was supported by the 7th Framework Program of the EU (AtheroRemo, grant agreement number 201668 and RiskyCAD, grant agreement number 305739). The work of W.M., M.E.K. and S.L. is supported as part of the Competence Cluster of Nutrition and Cardiovascular Health (nutriCARD) Halle-Jena-Leipzig (Germany) which is funded by the German Ministry of Education and Research (grant agreement numbers 01EA1808A and 01EA1411A). The authors thank the LURIC study team who were involved in patient recruitment as well as sample and data handling, in addition to the laboratory staff at the Ludwigshafen General Hospital and the universities of Freiburg, Ulm and Heidelberg, Germany.

Normative Aging Study

The Normative Aging Study (NAS) is a longitudinal study on aging established by the U.S. Department of Veterans Affairs in 1963. Details of the study have been published previously [1]. Briefly, the NAS is a closed cohort of 2,280 male veterans living in the Greater Boston area. Participants were enrolled after an initial health screening to determine whether they were free of known chronic medical conditions. Most of the participants were examined up to four times between 1999 and 2013. They have been reevaluated every three to five years on a continuous rolling basis using detailed on-site physical examinations and questionnaires. To control for the heterogeneity of race, a total 774 Caucasian participants aged 55-85 years at initial visit were used in the analysis. The molecular analyses in the US Department of Veterans Affairs (VA) Normative Aging Study have been supported by the U.S. National Institute of Environmental Health Sciences (NIEHS) (R01ES015172, R01ES021733). The VA Normative Aging Study is supported by the Cooperative Studies Program/ERIC, US Department of Veterans Affairs, and is a research component of the Massachusetts Veterans Epidemiology Research and Information Center (MAVERIC). Additional support to the VA Normative Aging Study was provided by the US Department of Agriculture, Agricultural Research Service (contract 53-K06-510). The views expressed in this paper are those of the authors and do not necessarily represent the views of the US Department of Veterans Affairs.

PIVUS The Prospective Investigation of the Vasculature in Uppsala Seniors (PIVUS) was initiated in 2001 as a prospective community-based cohort by inviting all persons aged 70 years and resident in Uppsala, Sweden, to participate (1,016 / 50.2% eligible were enrolled www.medsci.uu.se/pivus/). The PIVUS study was funded by Uppsala University Hospital.

Rhineland Study The Rhineland Study is an ongoing community-based cohort study in which all inhabitants of two geographically defined areas in the city of Bonn, Germany aged 30–100 years are being invited to participate. Persons living in these areas are predominantly German from Caucasian descent. Participation in the study is possible by invitation only. The only exclusion criterion is insufficient German language skills to give informed consent. The Rhineland Study's overarching aims are to investigate the etiology and prediction of age-related (neurodegenerative) diseases and to assess normal and pathological (brain) structure and function over the adult life course. The study started in 2016 and emphasizes deep phenotyping. Approval to undertake the Rhineland Study was obtained from the ethics committee of the University of Bonn, Medical Faculty. The study is carried out in accordance with the recommendations of the International Conference on Harmonization (ICH) Good Clinical Practice (GCP) standards (ICH-GCP). Written informed consent was obtained from all participants in accordance with the Declaration of Helsinki.

The Rhineland Study is supported by the German Center for Neurodegenerative Diseases (DZNE). The omics analyses in the Rhineland Study were partly supported by the Diet-Body-Brain Competence Cluster in Nutrition Research funded by the Federal Ministry of Education and Research (grant number 01EA1410C and FKZ: 01EA1809C).

RODAM The Research on Obesity and Diabetes among African Migrants (RODAM) study aims to unravel the underlying factors for the high prevalence of type 2 diabetes mellitus and obesity among Sub-Saharan African migrants with a focus on the interaction between environmental exposures and genetics. Between 2012 and 2015 Ghanaian adults (19–96 years of age) were recruited in rural Ghana, urban Ghana, Amsterdam, London and Berlin. Ghanaian origin was defined as being born in Ghana and having at least one parent born in Ghana or not born in Ghana but having both parents born in Ghana. In Ghana, the list of enumeration areas of the Ashanti region was used to as sampling frame to recruit participants in two cities (urban Ghana) and 15 villages (rural Ghana). In Amsterdam, Ghanaian participants were randomly drawn from the Amsterdam Municipal Register, which holds data on country of birth of citizens and their parents. In London, Ghanaian organizations served as the sampling frame as there was no population register for migrant groups. In Berlin, member lists of Ghanaian churches and organizations served as the sampling frame. The RODAM study is supported by the European Commission under the Framework Programme (grant number 278901).

The authors are grateful to the RODAM advisory board members for their valuable support in shaping the methods, to the research assistants, interviewers and other staff of the five research locations who have taken part in gathering the data and, most of all, to the Ghanaian volunteers participating in this project. We gratefully acknowledge J. van Straalen from the Department of Clinical Chemistry, Amsterdam University Medical Centers (Amsterdam, the Netherlands) for his valuable support with standardisation of the laboratory procedures, and the Academic Medical Center (AMC) Biobank for support in biobank management and storage of collected samples.

Rotterdam

The Rotterdam Study has been approved by the Medical Ethics Committee of the Erasmus MC (registration number MEC 02.1015) and by the Dutch Ministry of Health, Welfare and Sport (Population Screening Act WBO, license number 1071272-159521-PG). The Rotterdam Study has been entered into the Netherlands National Trial Register (NTR; www.trialregister.nl) and into the WHO International Clinical Trials Registry Platform (ICTRP; www.who.int/ictrp/network/primary/en/) under shared catalogue number NTR6831. All participants provided written informed consent to participate in the study and to have their information obtained from treating physicians. The generation and management of GWAS genotype data for the Rotterdam Study (RS I, RS II, RS III) was executed by the Human Genotyping Facility of the Genetic Laboratory of the Department of Internal Medicine, Erasmus MC, Rotterdam, The Netherlands. The GWAS datasets are supported by the Netherlands Organisation of Scientific Research NWO Investments (nr. 175.010.2005.011, 911-03-012), the Genetic Laboratory of the Department of Internal Medicine, Erasmus MC, the Research Institute for Diseases in the Elderly (014-93-015; RIDE2), the Netherlands Genomics Initiative (NGI)/Netherlands Organisation for Scientific Research (NWO) Netherlands Consortium for Healthy Aging (NCHA), project nr. 050-060-810. We thank Pascal Arp, Mila Jhamai, Marijn Verkerk, Lizbeth Herrera and Marjolein Peters, MSc, and Carolina Medina-Gomez, MSc, for their help in creating the GWAS database, and Karol Estrada, PhD, Yurii Aulchenko, PhD, and Carolina Medina-Gomez, MSc, for the creation and analysis of imputed data. The Rotterdam Study is funded by Erasmus Medical Center and Erasmus University, Rotterdam, Netherlands Organization for the Health Research and Development (ZonMw), the Research Institute for Diseases in the Elderly (RIDE), the Ministry of Education, Culture and Science, the Ministry for Health, Welfare and Sports, the European Commission (DG XII), and the Municipality of Rotterdam The authors are grateful to the study participants, the staff from the Rotterdam Study and the participating general practitioners and pharmacists. The generation and management of the Illumina 450K methylation array data (EWAS data) for the Rotterdam Study was executed by the Human Genotyping Facility of the Genetic Laboratory of the Department of Internal Medicine, Erasmus MC, the Netherlands. The EWAS data was funded by the Genetic Laboratory of the Department of Internal Medicine, Erasmus MC, and by the Netherlands Organization for Scientific Research (NWO; project number 184021007) and made available as a Rainbow Project (RP3; BIOS) of the Biobanking and Biomolecular Research Infrastructure Netherlands (BBMRI-NL). We thank Mr. Michael Verbiest, Ms. Mila Jhamai, Ms. Sarah Higgins, Mr. Marijn Verkerk, and Lisette Stolk for their help in creating the methylation database.

SHIP-Trend

SHIP is part of the Community Medicine Research net of the University of Greifswald, Germany, which is funded by the Federal Ministry of Education and Research (grants no. 01ZZ9603, 01ZZ0103, and 01ZZ0403), the Ministry of Cultural Affairs as well as the Social Ministry of the Federal State of Mecklenburg-West Pomerania, and the network 'Greifswald Approach to Individualized Medicine (GANI_MED)' funded by the Federal Ministry of Education and Research (grant 03IS2061A). DNA methylation data have been supported by the DZHK (grant 81X3400104). The University of Greifswald is a member of the Caché Campus program of the InterSystems GmbH. The SHIP authors are grateful to Paul S. DeVries for his support with the EWAS pipeline.

SKIPOGH

The Swiss Kidney Project on Genes in Hypertension (SKIPOGH) is a multicenter family-based cohort study exploring the genetic determinants of blood pressure and kidney function in the general adult population of Switzerland as previously described in detail [Moulin, 2017 PMID 28888328]. All participants gave their informed consent and the SKIPOGH study was approved by the ethics committees of the University Hospital of Lausanne, Geneva University Hospital and Bern University Hospital. The SKIPOGH study is supported by a grant from the Swiss national science foundation (FN33CM30-124087).

YFS

The Young Finns Study has been financially supported by the Academy of Finland: grants 322098, 286284, 134309 (Eye), 126925, 121584, 124282, 129378 (Salve), 117787 (Gendi), and 41071 (Skidi); the Social Insurance Institution of Finland; Competitive State Research Financing of the Expert Responsibility area of Kuopio, Tampere and Turku University Hospitals (grant X51001); Juho Vainio Foundation; Paavo Nurmi Foundation; Finnish Foundation for Cardiovascular Research ; Finnish Cultural Foundation; The Sigrid Juselius Foundation; Tampere Tuberculosis Foundation; Emil Aaltonen Foundation; Yrjö Jahnsson Foundation; Signe and Ane Gyllenberg Foundation; Diabetes Research Foundation of Finnish Diabetes Association; EU Horizon 2020 (grant 755320 for TAXINOMISIS); This project has received funding from the European Union's Horizon 2020 research and innovation programme under grant agreement No 848146; European Research Council (grant 742927 for MULTIEPIGEN project); Tampere University Hospital Supporting Foundation and Finnish Society of Clinical Chemistry.

Group Authors and Other funding acknowledgments

The work of O.M.W. was supported by NIDDK grant R01DK114091 and V.L.H.K. by NIDDK grant F32DK124960. A.A.A. and K.A.C.M. are supported by the Intramural Research Program of the National Institutes of Health in the Center for Research on Genomics and Global Health (CRGGH). The CRGGH is supported by the National Human Genome Research Institute, the National Institute of Diabetes and Digestive and Kidney Diseases, the Center for Information Technology, and the Office of the Director at the National Institutes of Health (1ZIAHG200362).

The Genetics of DNA Methylation Consortium

Josine L. Min is supported by the UK Medical Research Council Integrative Epidemiology Unit at the University of Bristol (MC_UU_00011/5).
Gibran Hemani is funded by the Wellcome Trust and the Royal Society (208806/Z/17/Z).

The Estonian Biobank Research Team: group author acknowledgment

Tõnu Esko[98], Andres Metspalu[98], Reedik Mägi[98], Lili Milani[58] Mari Nelis[98]
[98]Estonian Biobank, Institute of Genomics, University of Tartu, Tartu, Estonia

Supplementary Note 2: Results on the reverse Mendelian randomization (MR) analysis of the causal effect of serum urate on DNA methylation levels

The reverse MR analysis using all available index SNPs of serum urate among persons of European ancestry resulted in five significant CpGs (3 in the *SLC2A9* region). Leave-one-out analysis showed that the significant causal effects were driven by the index SNP at *SLC2A9*, rs4447862 (**Supplementary Figures 17A to 17E**) and no longer significant upon its removal. Therefore, the significant results including rs4447862 were associations driven by this SNP, rather than causal effects of serum urate on DNA methylation levels.

Supplementary Note 3: Minimum detectable effect size in forward MR analysis of DNA methylation on serum urate or gout.

		All meQTLs in primary analysis		Excluding meQTLs with r^2 >0.05 with 5 urate SNPs in EA*	
**Outcome**	**CpG**	**# of meQTLs**	**Min detectable effect size****	**# of meQTLs**	**Min detectable effect size****
Urate	cg02387843	6	0.044	Not enough meQTL for MR analysis (<4)	
Urate	cg13841979	10	0.039	7	0.104
Urate	cg03725404	8	0.047	Not enough meQTL for MR analysis (<4)	
Urate	cg11266682	11	0.020		
Gout	cg03725404	8	0.918	Not enough meQTL for MR analysis (<4)	

*The 5 urate SNPs in *SLC2A9* in EA reported in Tin *et al*. 2019: SNP with lowest p-value in EA: rs4447862, independent SNPs identified by GCTA stepwise selection: rs6825187, rs62286563, rs10017305, rs73224492.
** The effect size was estimated for mg/dL per SD of rank-based transformed DNA methylation beta value for urate and OR per SD of rank-based transformed DNA methylation beta value for gout.
Assumptions: 90% power, significance threshold: 1.9E-3, sample sizes based on those used in the MR analysis: GoDMC meQTL (n=27,750), serum urate GWAS (n=288,649).

References

1. Burgess, S., Butterworth, A. & Thompson, S.G. Mendelian randomization analysis with multiple genetic variants using summarized data. *Genet Epidemiol* **37**, 658-65 (2013).

2. Burgess, S. *et al.* Guidelines for performing Mendelian randomization investigations [version 2; peer review: 2 approved]. *Wellcome Open Research* **4**(2020).

Supplementary Figures 1 to 17

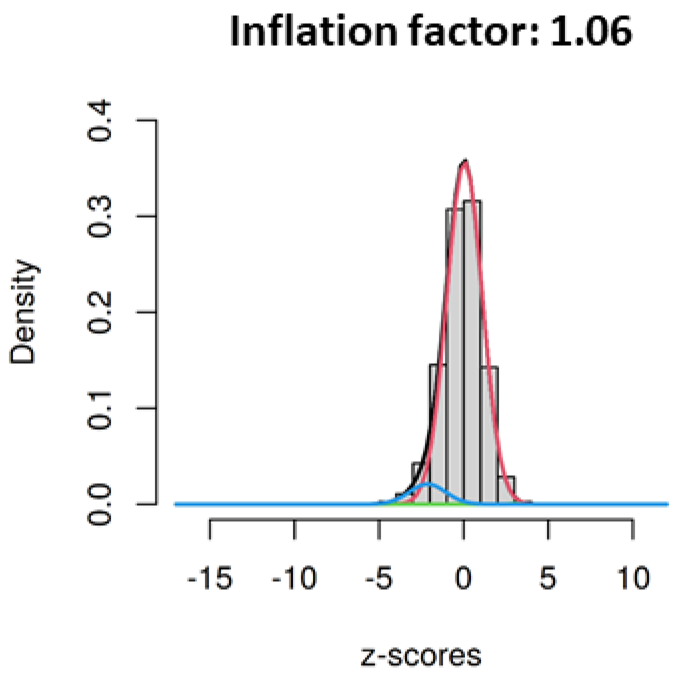

Supplementary Figure 1. Distribution of the z-scores of the meta-analysis combining discovery and replication results. The colored lines represent the three-component normal mixture fitted as estimated by the Bayesian BACON method: the red line the fit of the empirical null distribution, and the blue and green lines the estimated fits of the distribution of negative and positive associations, respectively. The black line represents the overall fit of the z-scores.

Supplementary Figures 2A to 2AF. Forest plots by ancestry of DNA methylation beta value per mg/dL of serum urate on 32 replicated CpGs with heterogeneity >50% from the meta-analysis of four ancestry groups: European (17 studies), African American (5 studies), and one study each for South Asian and Sub-Saharan Africa. Among these CpGs, 90% (29 CpGs) had consistent effect directions across the ancestry groups. Given that only one study each was available for South Asian and Sub-Saharan African ancestry, some heterogeneity might reflect study heterogeneity. The CpGs are ordered by chromosomal position. Abbreviations: EA: European ancestry, AA: African American, SA: South Asian, AFR: Sub-Saharan Africa. The numbers in parentheses next to the ancestry abbreviations reflect sample size. The I^2 statistic is provided as a measure of heterogeneity across the four groups. Whiskers correspond to 95% confidence intervals. Sample size for each plot: A (cg23913963, n=15,966), B (cg03387723, n=17,987), C (cg06983052, n=17,446), D (cg03725309, n=15,975), E (cg18660898, n=16,517), F (cg16375541, n=17,441), G (cg12825509, n=15,964), H (cg21795255, n=15,983), I (cg20479063, n=15,942), J (cg02734326, n=17,419), K (cg00071950, n=17,452), L (cg11266682, n=17,447), M (cg14348967, n=17,396), N (cg06690548, n=17,921), O (cg05201185, n=15,257), P (cg27486585, n=17,440), Q (cg20813374, n=17,433), R (cg26669717, n=17,432), S (cg00422488, n=17,410), T (cg13823169, n=17,445), U (cg19939077, n=17,452), V (cg06178669, n=15,818), W (cg18125510, n=17,434), X (cg04625615, n=17,441), Y (cg23684449, n=17,441), Z (cg05288253, n=15,981), AA (cg26043149, n=17,426), AB (cg06599169, n=17,440), AC (cg02711608, n=17,449), AD (cg21766592, n=17,444), AE (cg00711496, n=17,438), AF (cg18405341, n=17,444).

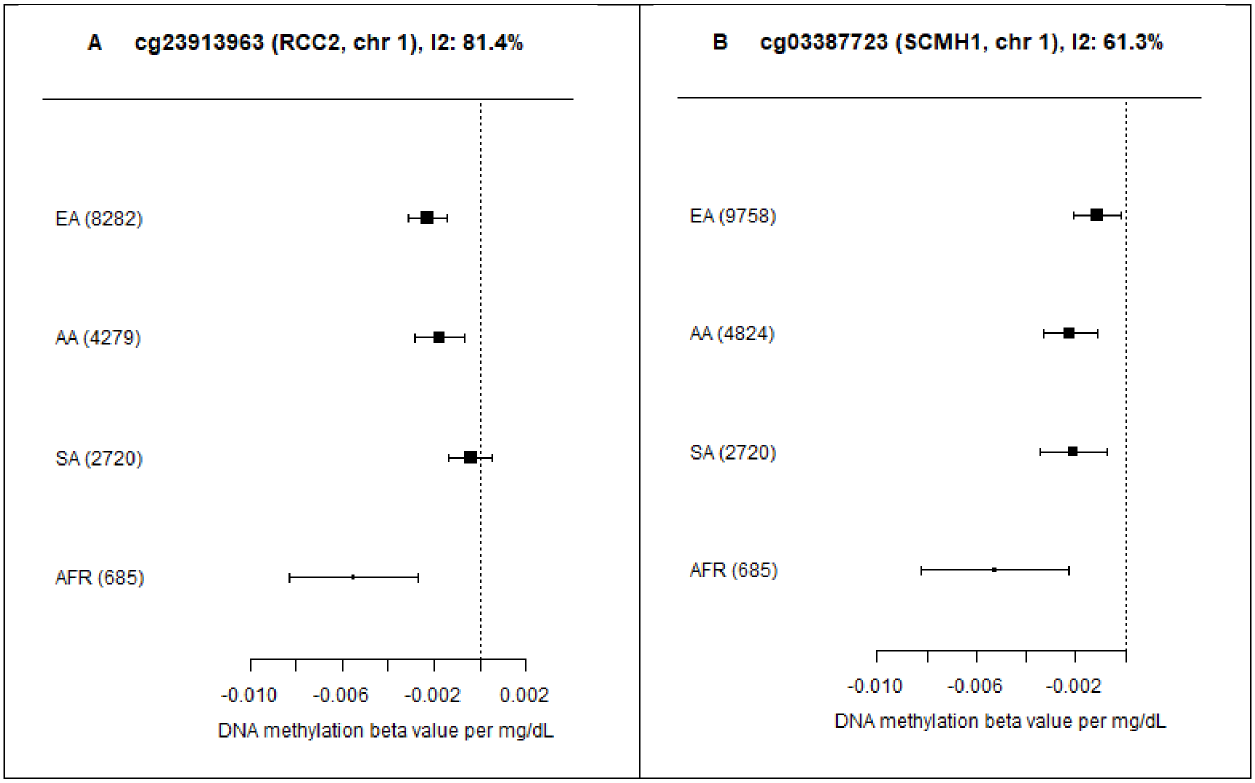

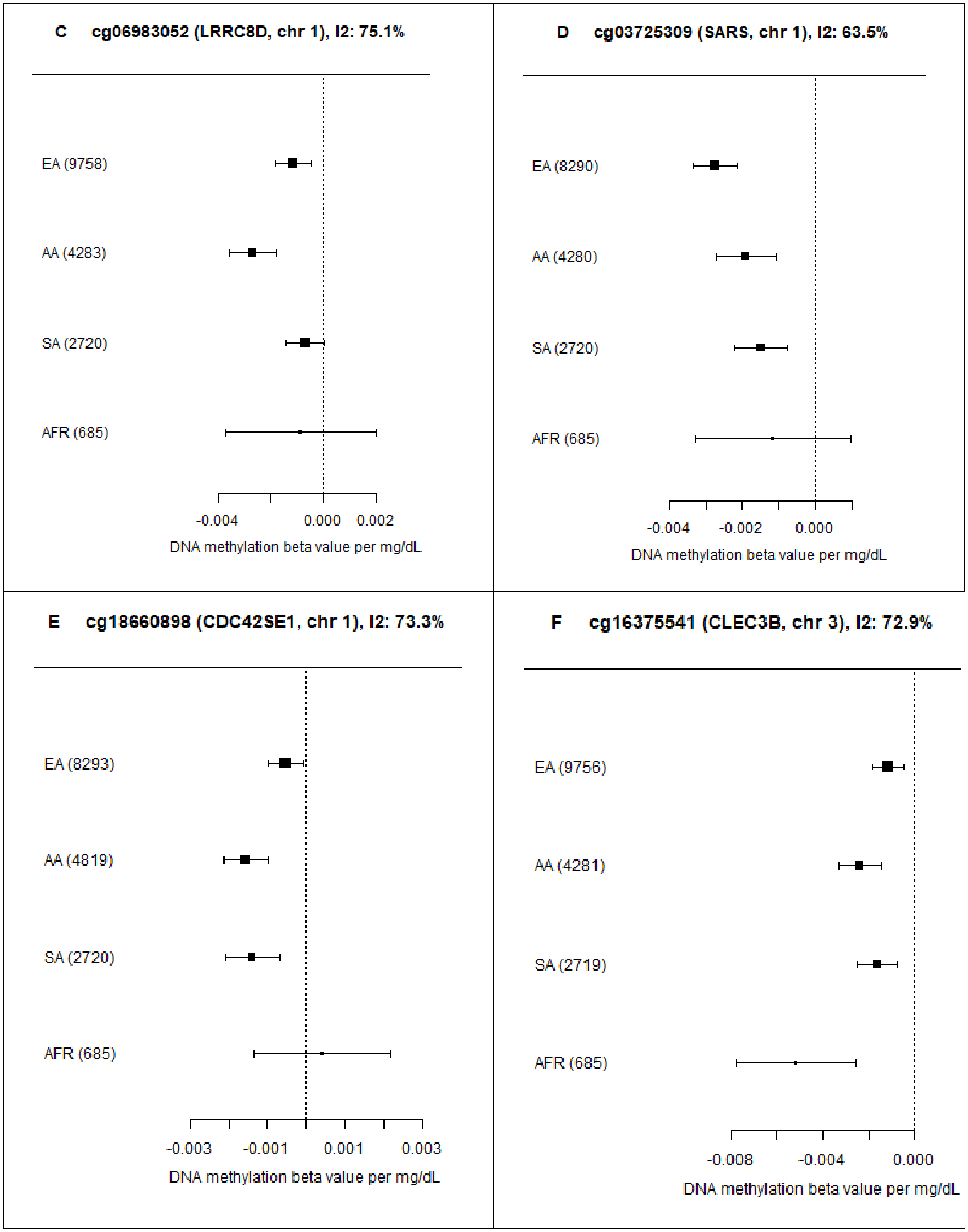
C cg06983052 (LRRC8D, chr 1), I2: 75.1%
EA (9758)
AA (4283)
SA (2720)
AFR (685)
-0.004
0.000
0.002
DNA methylation beta value per mg/dL
D cg03725309 (SARS, chr 1), I2: 63.5%
EA (8290)
AA (4280)
SA (2720)
AFR (685)
-0.004
-0.002
0.000
DNA methylation beta value per mg/dL
E cg18660898 (CDC42SE1, chr 1), I2: 73.3%
EA (8293)
AA (4819)
SA (2720)
AFR (685)
-0.003
-0.001
0.001
0.003
DNA methylation beta value per mg/dL
F cg16375541 (CLEC3B, chr 3), I2: 72.9%
EA (9756)
AA (4281)
SA (2719)
AFR (685)
-0.008
-0.004
0.000
DNA methylation beta value per mg/dL

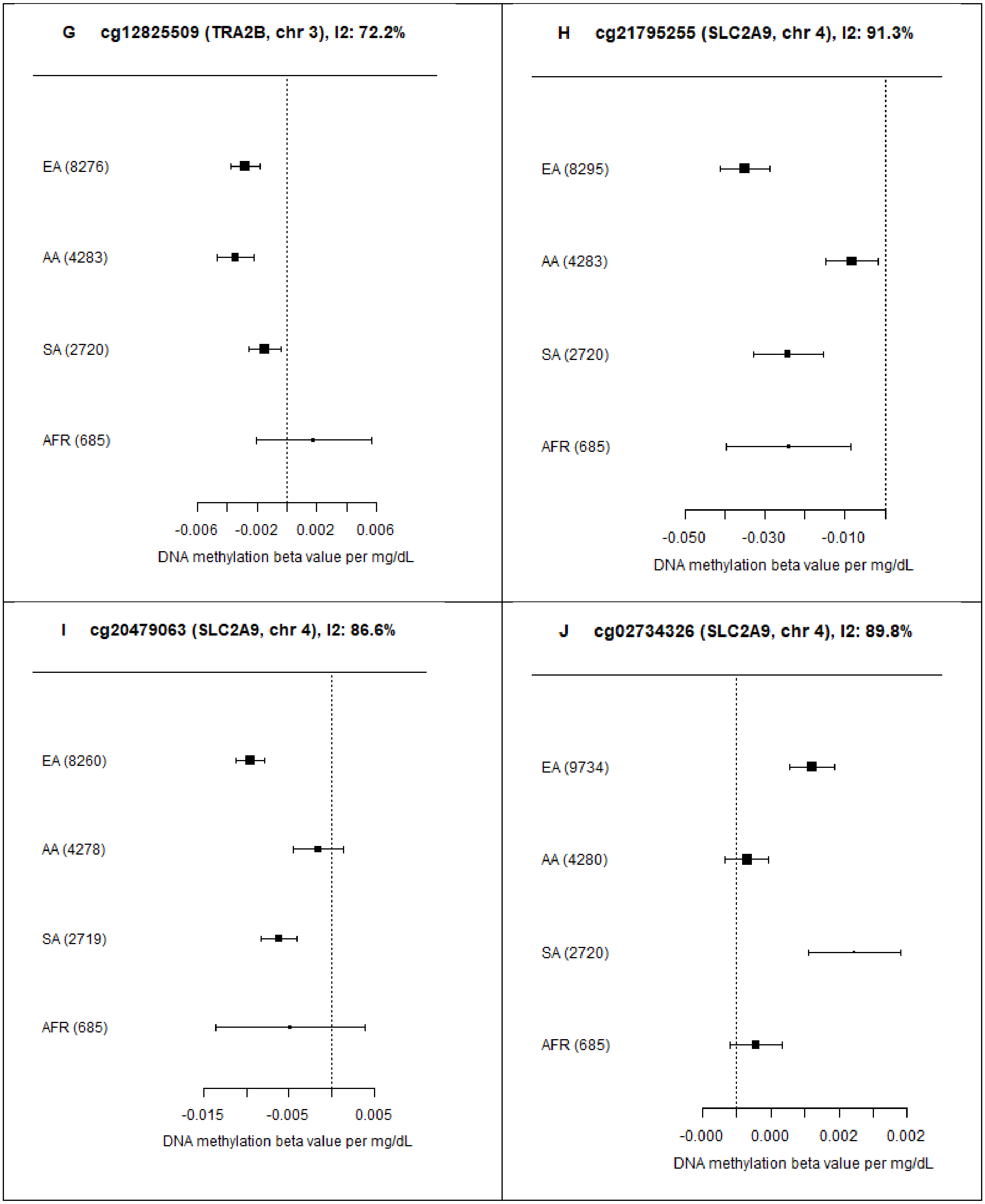
G cg12825509 (TRA2B, chr 3), I2: 72.2%
EA (8276)
AA (4283)
SA (2720)
AFR (685)
-0.006
-0.002
0.002
0.006
DNA methylation beta value per mg/dL
H cg21795255 (SLC2A9, chr 4), I2: 91.3%
EA (8295)
AA (4283)
SA (2720)
AFR (685)
-0.050
-0.030
-0.010
DNA methylation beta value per mg/dL
I cg20479063 (SLC2A9, chr 4), I2: 86.6%
EA (8260)
AA (4278)
SA (2719)
AFR (685)
-0.015
-0.005
0.005
DNA methylation beta value per mg/dL
J cg02734326 (SLC2A9, chr 4), I2: 89.8%
EA (9734)
AA (4280)
SA (2720)
AFR (685)
-0.000
0.000
0.002
0.002
DNA methylation beta value per mg/dL

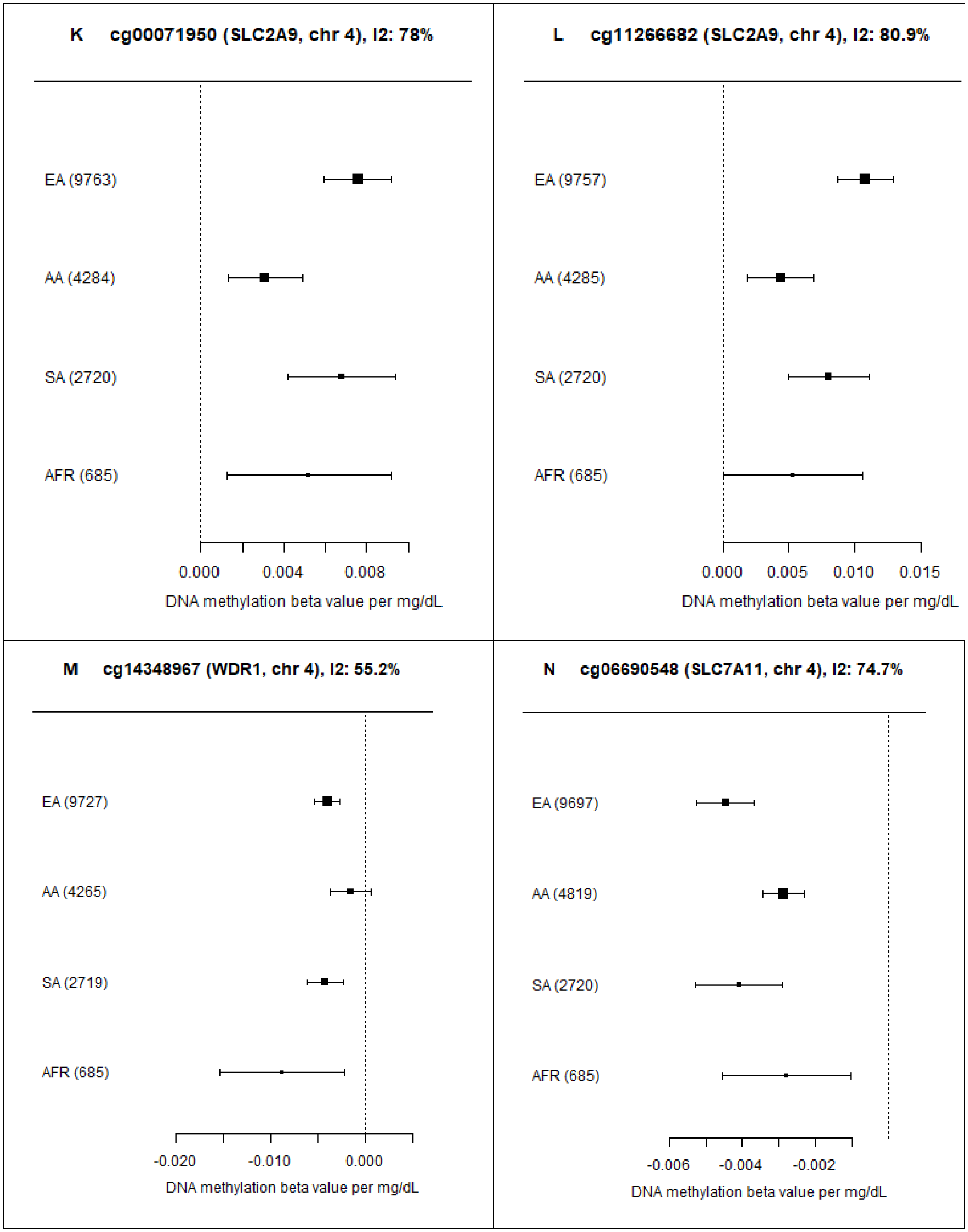
K cg00071950 (SLC2A9, chr 4), I2: 78%
EA (9763)
AA (4284)
SA (2720)
AFR (685)
0.000
0.004
0.008
DNA methylation beta value per mg/dL
L cg11266682 (SLC2A9, chr 4), I2: 80.9%
EA (9757)
AA (4285)
SA (2720)
AFR (685)
0.000
0.005
0.010
0.015
DNA methylation beta value per mg/dL
M cg14348967 (WDR1, chr 4), I2: 55.2%
EA (9727)
AA (4265)
SA (2719)
AFR (685)
-0.020
-0.010
0.000
DNA methylation beta value per mg/dL
N cg06690548 (SLC7A11, chr 4), I2: 74.7%
EA (9697)
AA (4819)
SA (2720)
AFR (685)
-0.006
-0.004
-0.002
DNA methylation beta value per mg/dL

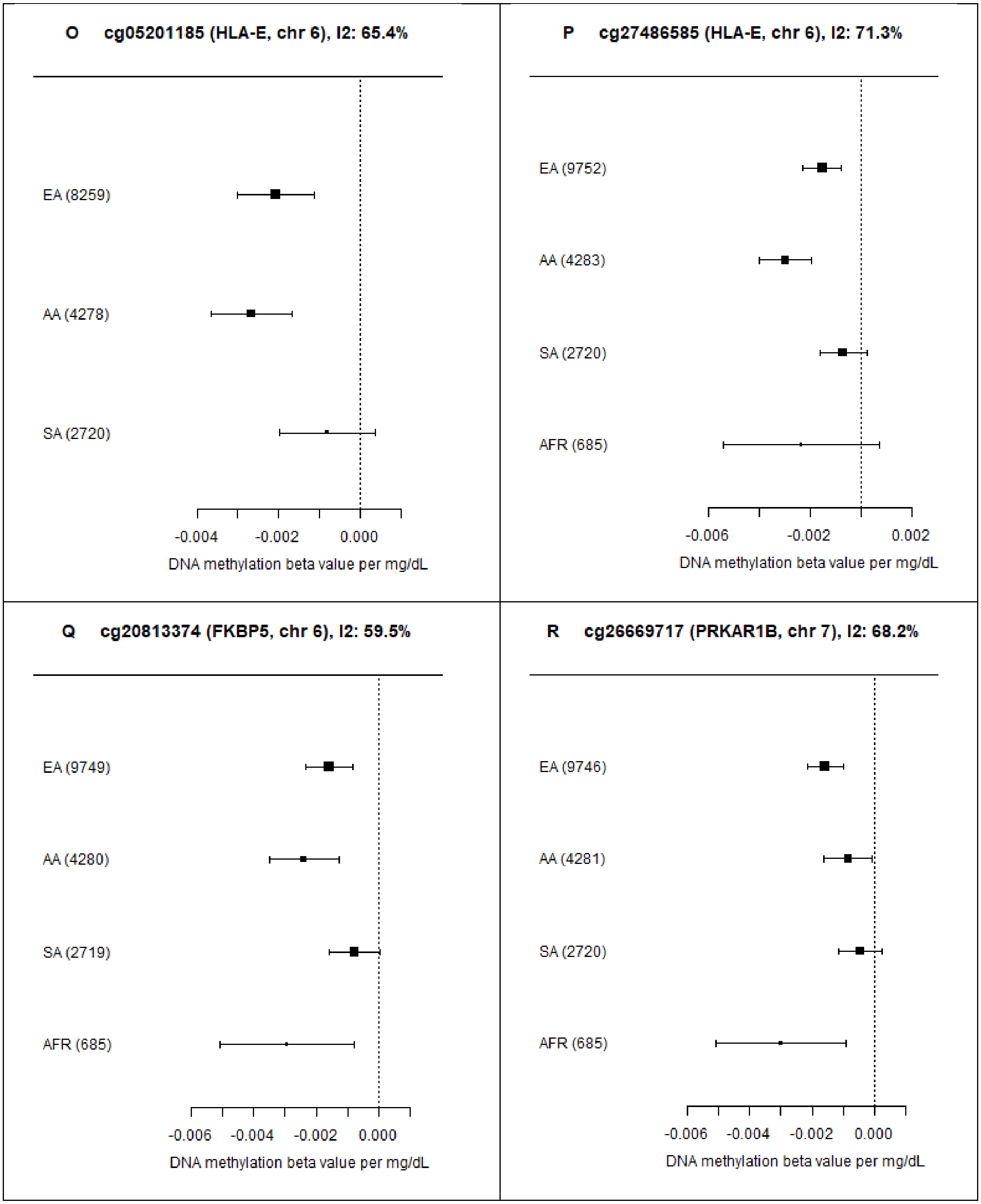
O cg05201185 (HLA-E, chr 6), I2: 65.4%
EA (8259)
AA (4278)
SA (2720)
-0.004
-0.002
0.000
DNA methylation beta value per mg/dL
P cg27486585 (HLA-E, chr 6), I2: 71.3%
EA (9752)
AA (4283)
SA (2720)
AFR (685)
-0.006
-0.002
0.002
DNA methylation beta value per mg/dL
Q cg20813374 (FKBP5, chr 6), I2: 59.5%
EA (9749)
AA (4280)
SA (2719)
AFR (685)
-0.006
-0.004
-0.002
0.000
DNA methylation beta value per mg/dL
R cg26669717 (PRKAR1B, chr 7), I2: 68.2%
EA (9746)
AA (4281)
SA (2720)
AFR (685)
-0.006
-0.004
-0.002
0.000
DNA methylation beta value per mg/dL

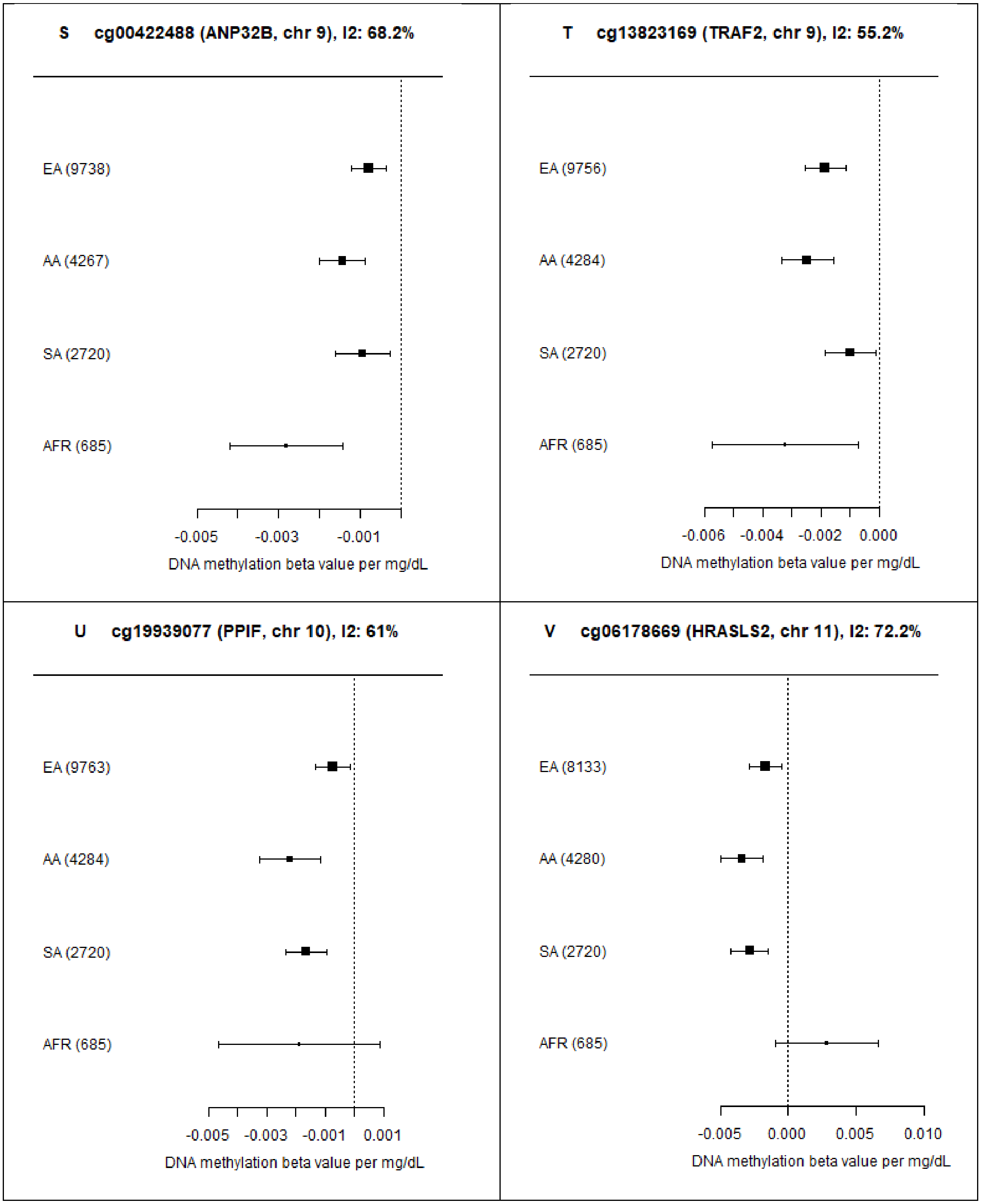
S cg00422488 (ANP32B, chr 9), I2: 68.2%
EA (9738)
AA (4267)
SA (2720)
AFR (685)
-0.005 -0.003 -0.001
DNA methylation beta value per mg/dL
T cg13823169 (TRAF2, chr 9), I2: 55.2%
EA (9756)
AA (4284)
SA (2720)
AFR (685)
-0.006 -0.004 -0.002 0.000
DNA methylation beta value per mg/dL
U cg19939077 (PPIF, chr 10), I2: 61%
EA (9763)
AA (4284)
SA (2720)
AFR (685)
-0.005 -0.003 -0.001 0.001
DNA methylation beta value per mg/dL
V cg06178669 (HRASLS2, chr 11), I2: 72.2%
EA (8133)
AA (4280)
SA (2720)
AFR (685)
-0.005 0.000 0.005 0.010
DNA methylation beta value per mg/dL

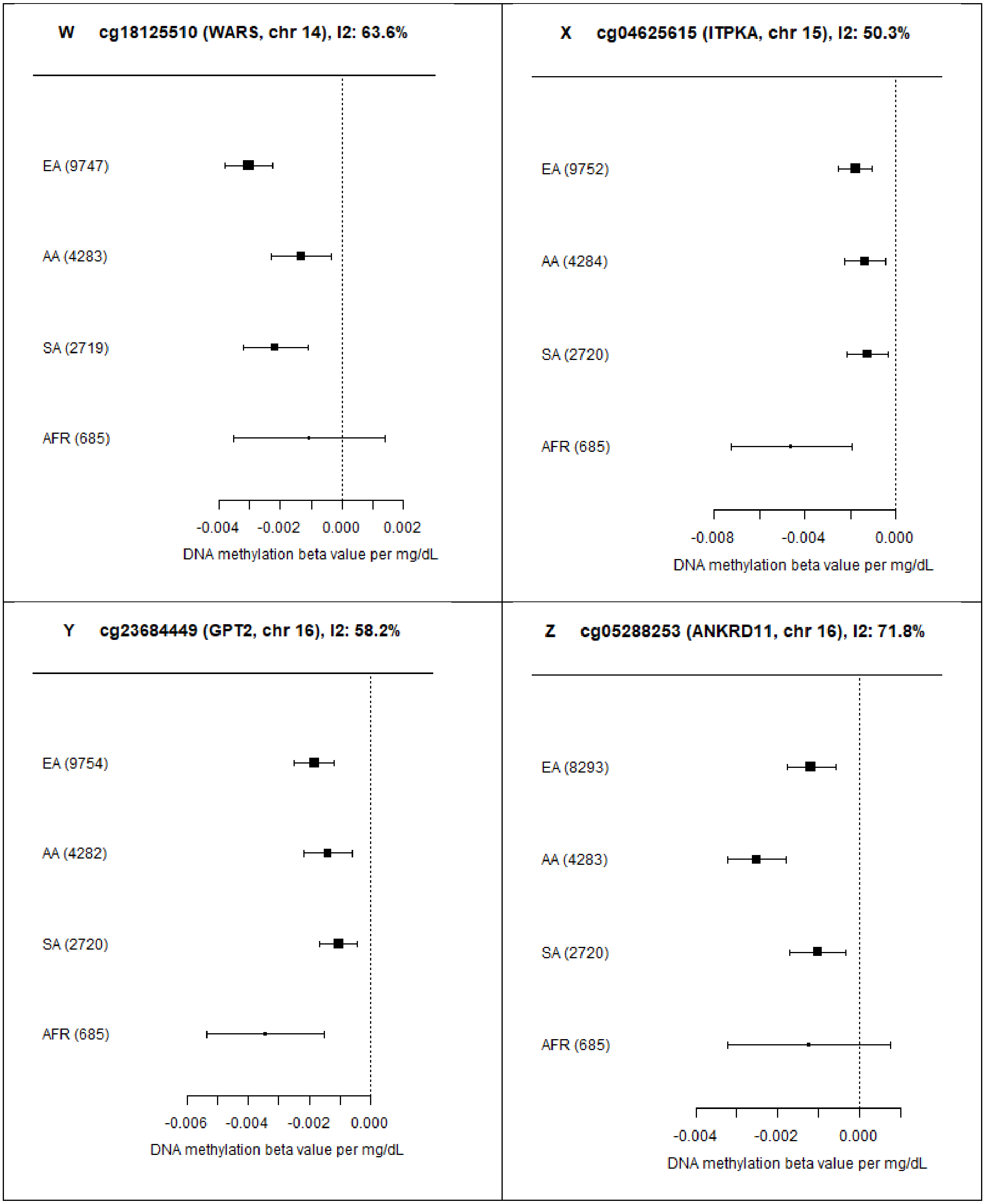
W cg18125510 (WARS, chr 14), I2: 63.6%
EA (9747)
AA (4283)
SA (2719)
AFR (685)
-0.004
-0.002
0.000
0.002
DNA methylation beta value per mg/dL
X cg04625615 (ITPKA, chr 15), I2: 50.3%
EA (9752)
AA (4284)
SA (2720)
AFR (685)
-0.008
-0.004
0.000
DNA methylation beta value per mg/dL
Y cg23684449 (GPT2, chr 16), I2: 58.2%
EA (9754)
AA (4282)
SA (2720)
AFR (685)
-0.006
-0.004
-0.002
0.000
DNA methylation beta value per mg/dL
Z cg05288253 (ANKRD11, chr 16), I2: 71.8%
EA (8293)
AA (4283)
SA (2720)
AFR (685)
-0.004
-0.002
0.000
DNA methylation beta value per mg/dL

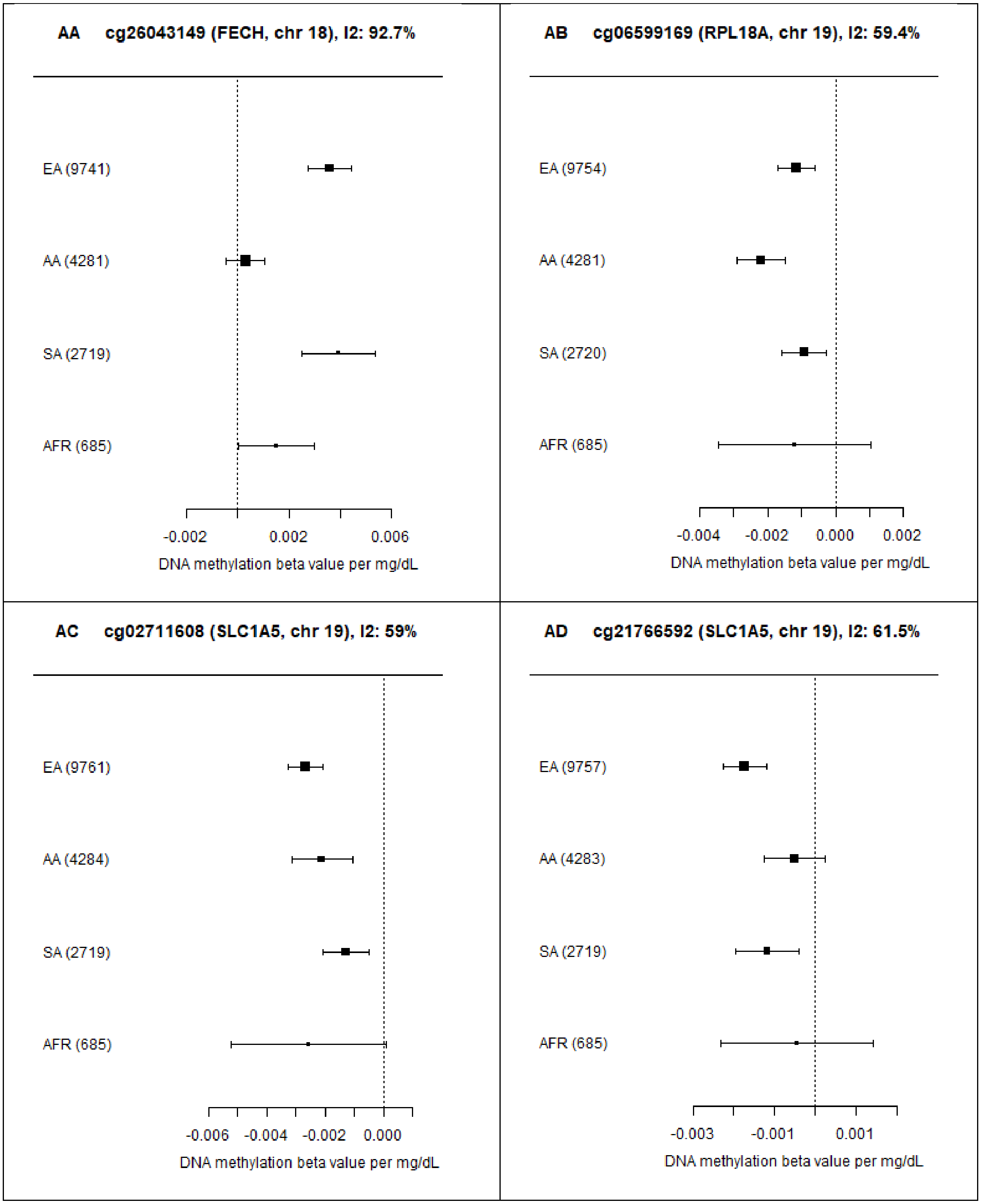
AA cg26043149 (FECH, chr 18), I2: 92.7%
EA (9741)
AA (4281)
SA (2719)
AFR (685)
-0.002
0.002
0.006
DNA methylation beta value per mg/dL
AB cg06599169 (RPL18A, chr 19), I2: 59.4%
EA (9754)
AA (4281)
SA (2720)
AFR (685)
-0.004
-0.002
0.000
0.002
DNA methylation beta value per mg/dL
AC cg02711608 (SLC1A5, chr 19), I2: 59%
EA (9761)
AA (4284)
SA (2719)
AFR (685)
-0.006
-0.004
-0.002
0.000
DNA methylation beta value per mg/dL
AD cg21766592 (SLC1A5, chr 19), I2: 61.5%
EA (9757)
AA (4283)
SA (2719)
AFR (685)
-0.003
-0.001
0.001
DNA methylation beta value per mg/dL

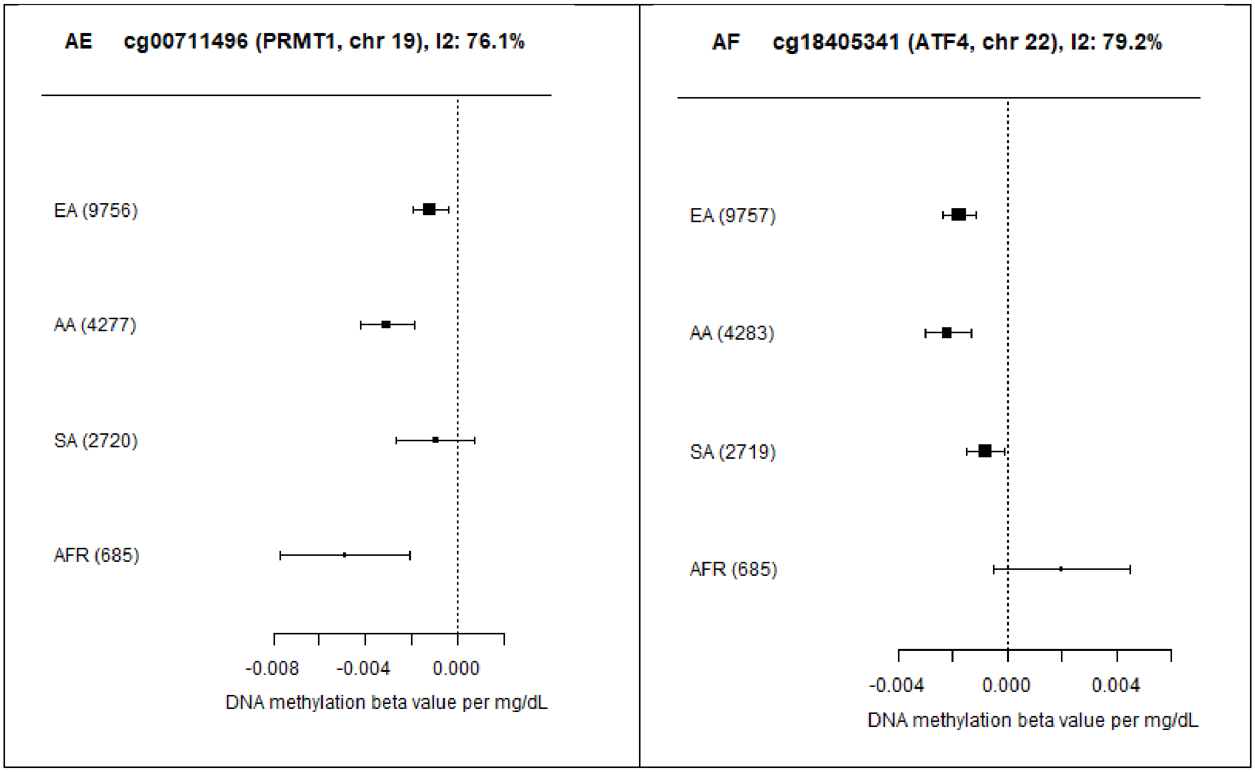
AE cg00711496 (PRMT1, chr 19), I2: 76.1%
EA (9756)
AA (4277)
SA (2720)
AFR (685)
-0.008
-0.004
0.000
DNA methylation beta value per mg/dL
AF cg18405341 (ATF4, chr 22), I2: 79.2%
EA (9757)
AA (4283)
SA (2719)
AFR (685)
-0.004
0.000
0.004
DNA methylation beta value per mg/dL

Supplementary Figure 3. Scatter plots of the effect size of the meQTLs on DNA methylation levels and serum urate for the four CpGs with significant causal effect estimates on serum urate: cg02387843 (A), cg03725404 (B), cg11266682 (C), and cg13841979 (D). X-axis is the SNP effect on the CpG in SD of rank-based transformed DNA methylation beta value per allele, and the y-axis is the SNP effect on the outcome, serum urate, in mg/dL per allele. The whiskers represent 95% confidence intervals. Sample size: GoDMC meQTL study n=27,750), serum urate GWAS (n=288,649). Abbreviations: MR, Mendelian randomization; SNP, single nucleotide polymorphism; SD, standard deviation.

Supplementary Figure 3A

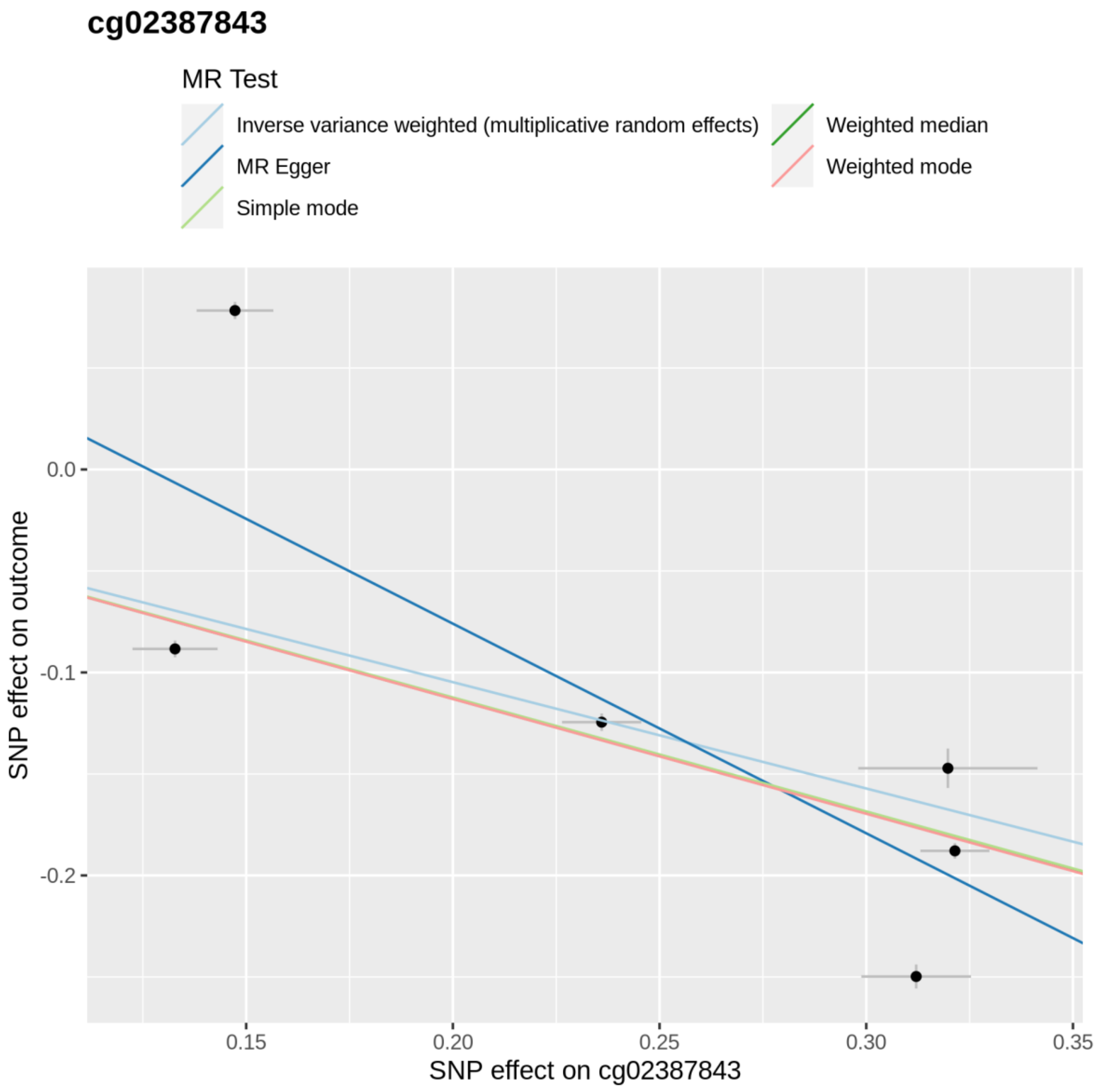

Supplementary Figure 3B

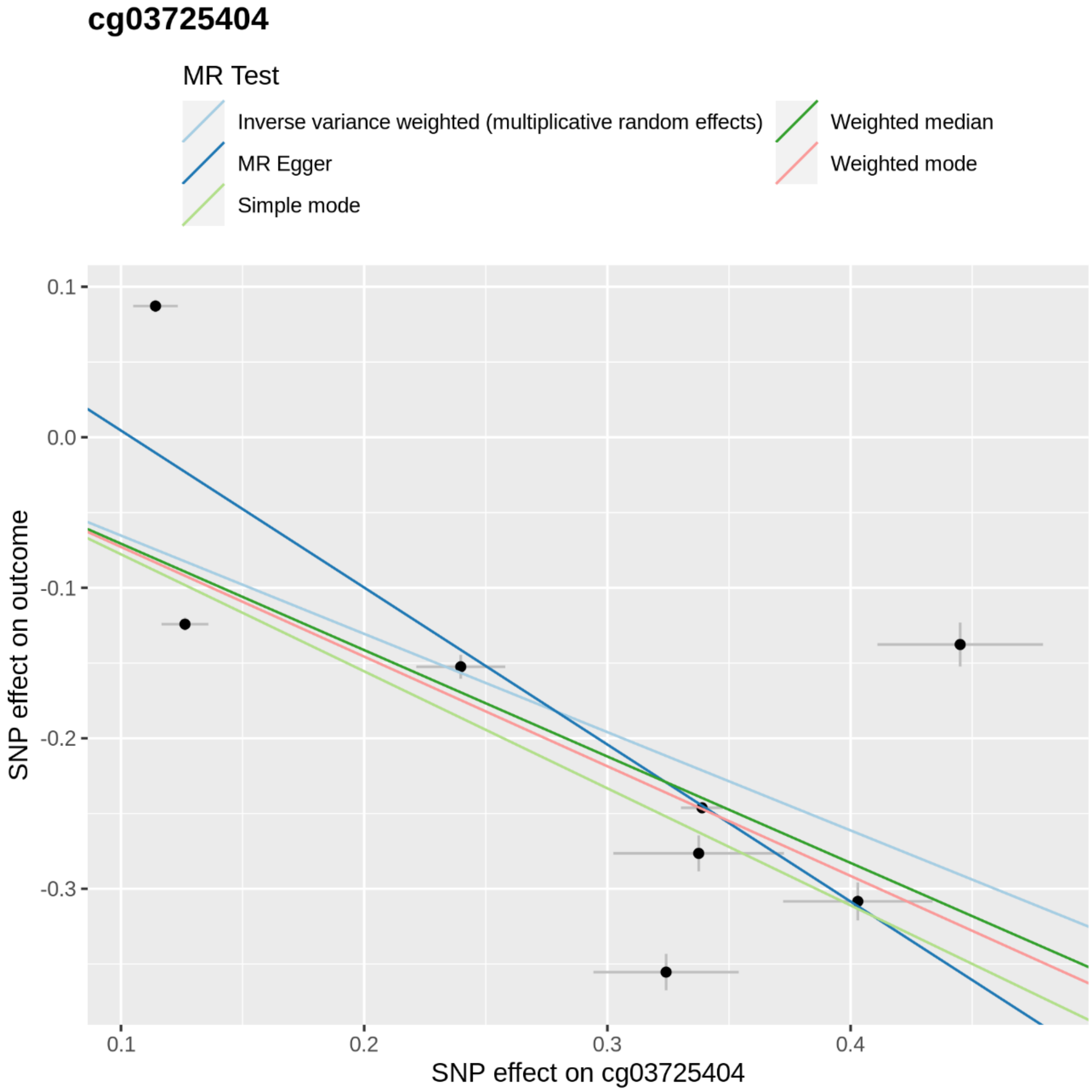

Supplementary Figure 3C

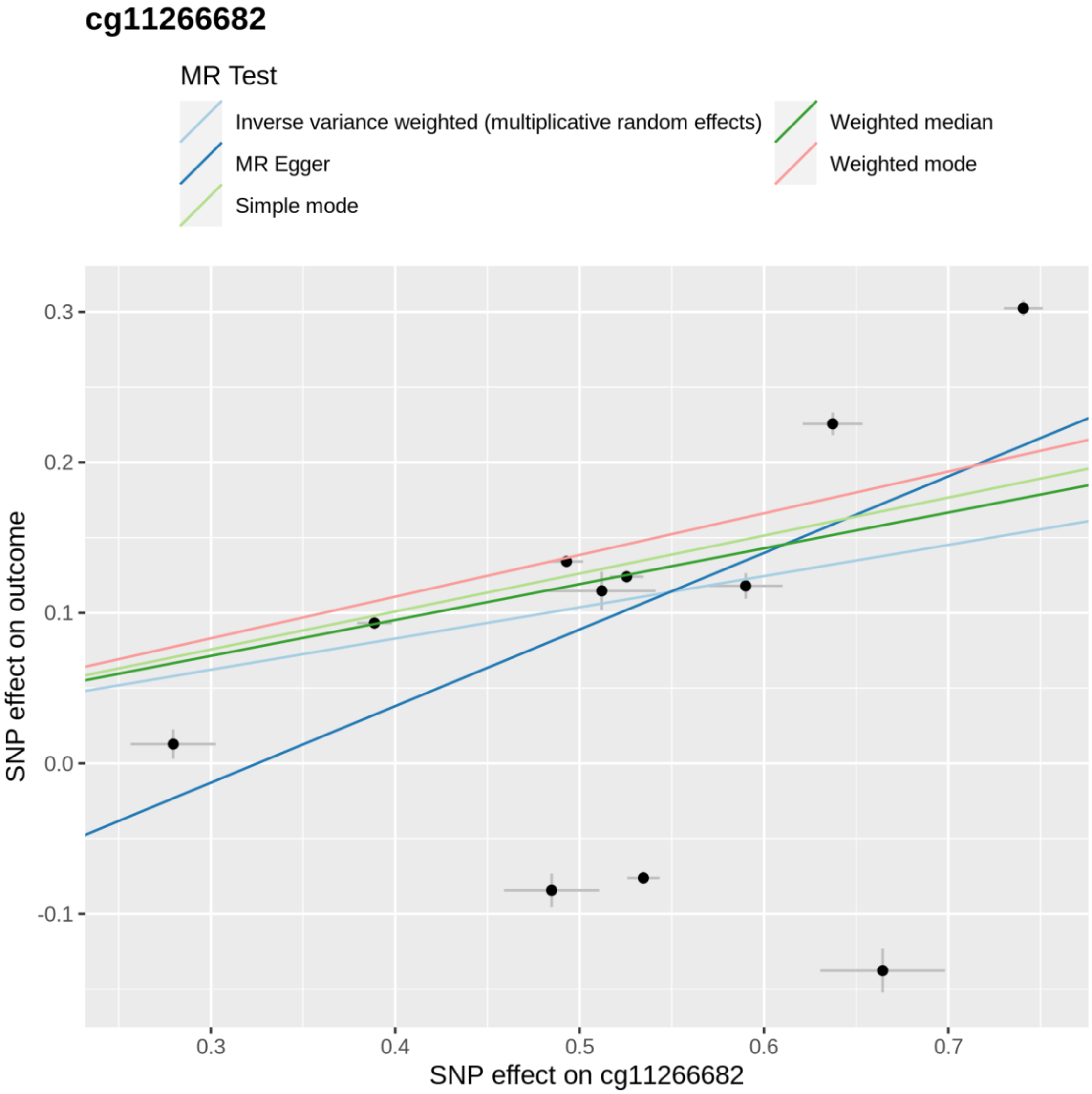

Supplementary Figure 3D

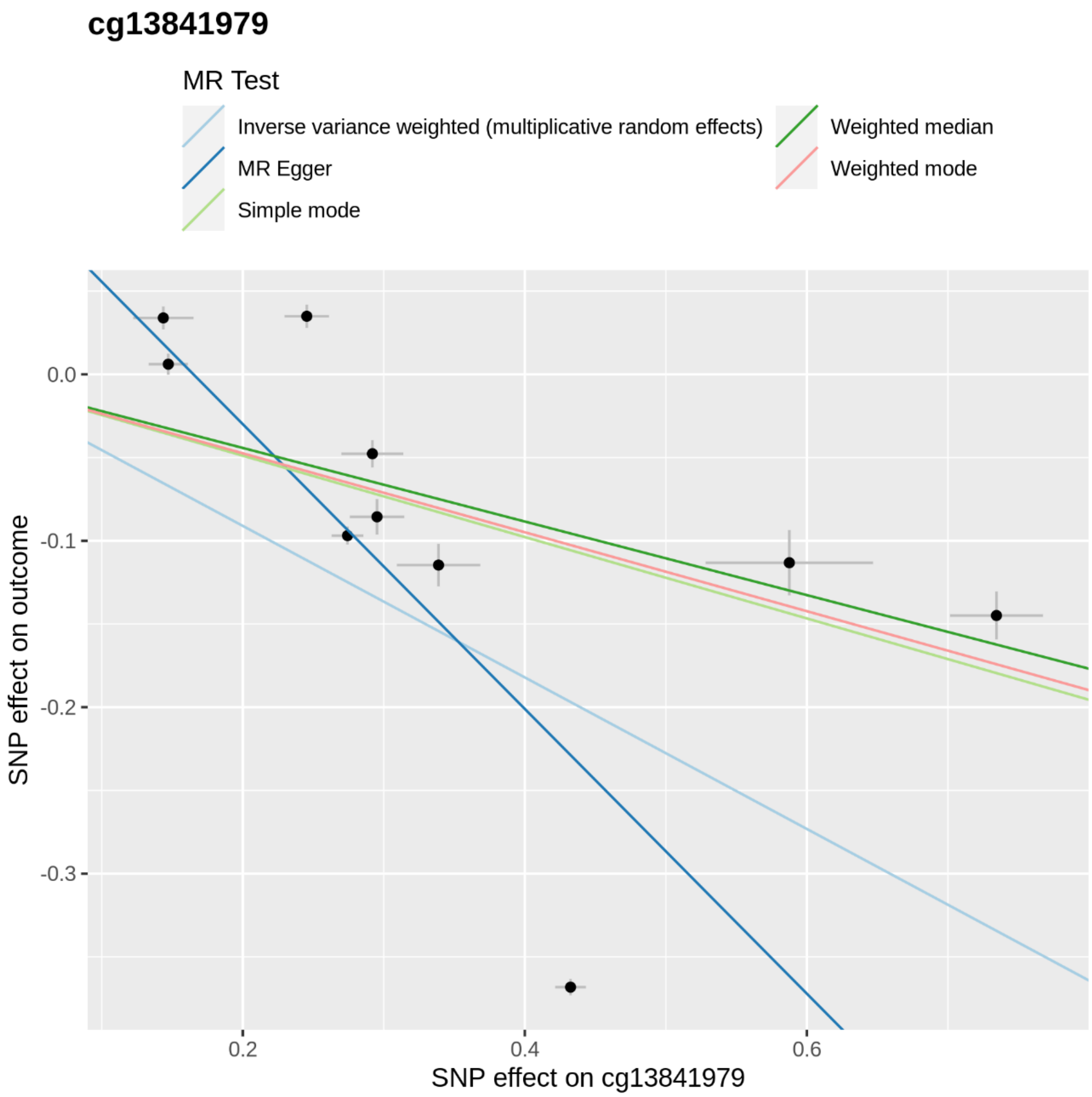

Supplementary Figure 4. Scatter plots of the effect size of meQTLs on DNA methylation levels and gout for the one CpG with significant causal effect estimates on gout. X-axis is the SNP effect on CpG in SD of rank-based transformed DNA methylation beta value per allele, and the y-axis is the SNP effect on gout as the outcome in log(odds ratio) per allele. The whiskers represent 95% confidence intervals. Samle size: GoDMC meQTL study (n=27,750), gout GWAS (n=692,537). Abbreviations: MR, Mendelian randomization; SNP, single nucleotide polymorphism; SD, standard deviation.

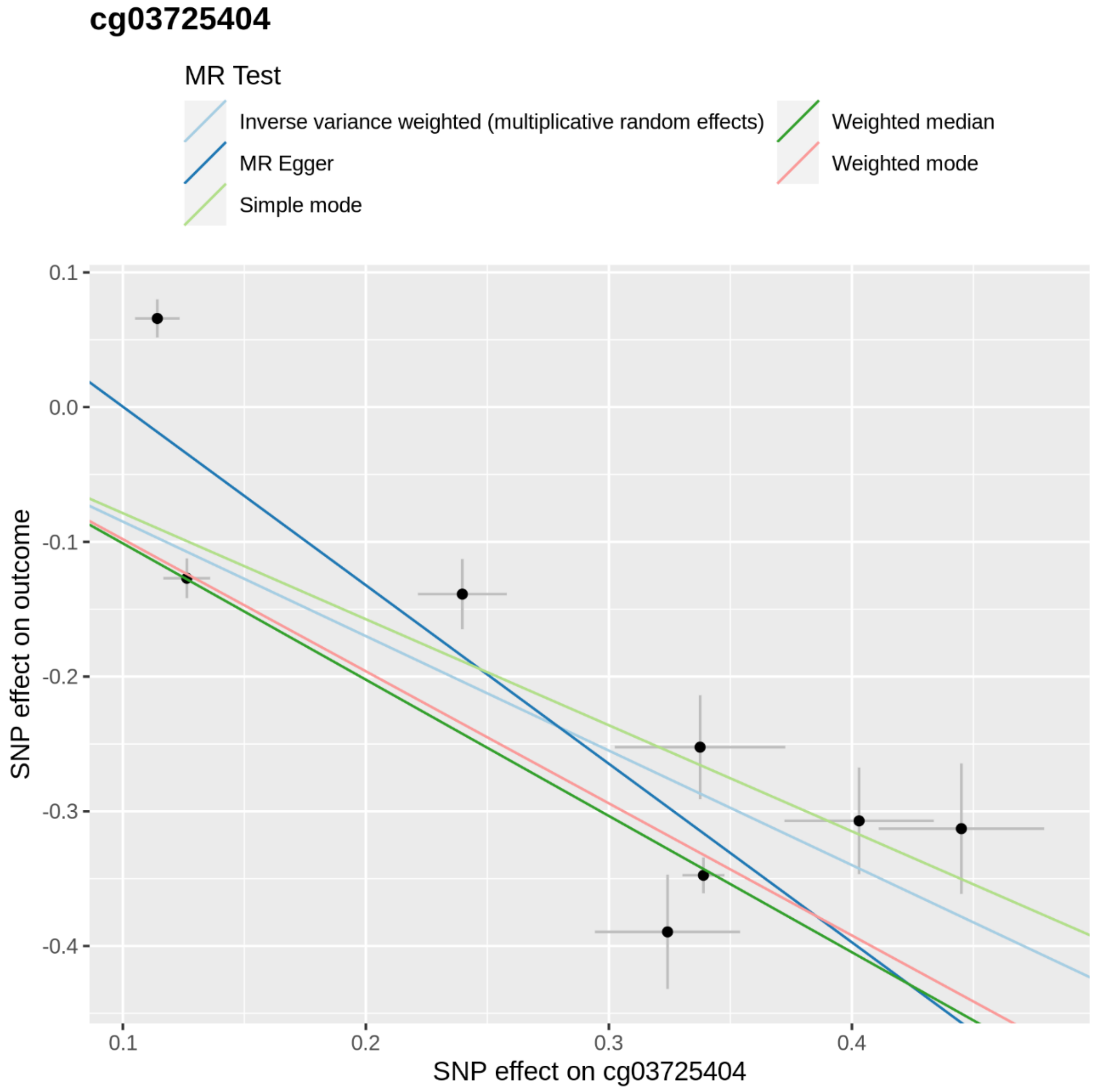

Supplementary Figure 5. For the four CpGs with significant causal effects on serum urate, plots of the association p-value of the meQTLs (association with DNA methylation) included in the Mendelian randomization analysis (top plot) and r^2 among meQTLs (bottom plot): cg02387843 (A), cg03725404 (B), cg11266682 (C), and cg13841979 (D). The p-values were 2-sided and obtained from inverse variance weighted fixed effect meta-analysis. Sample size: GoDMC meQTL study (n=27,750), r^2 (n=804).

Supplementary Figure 5A

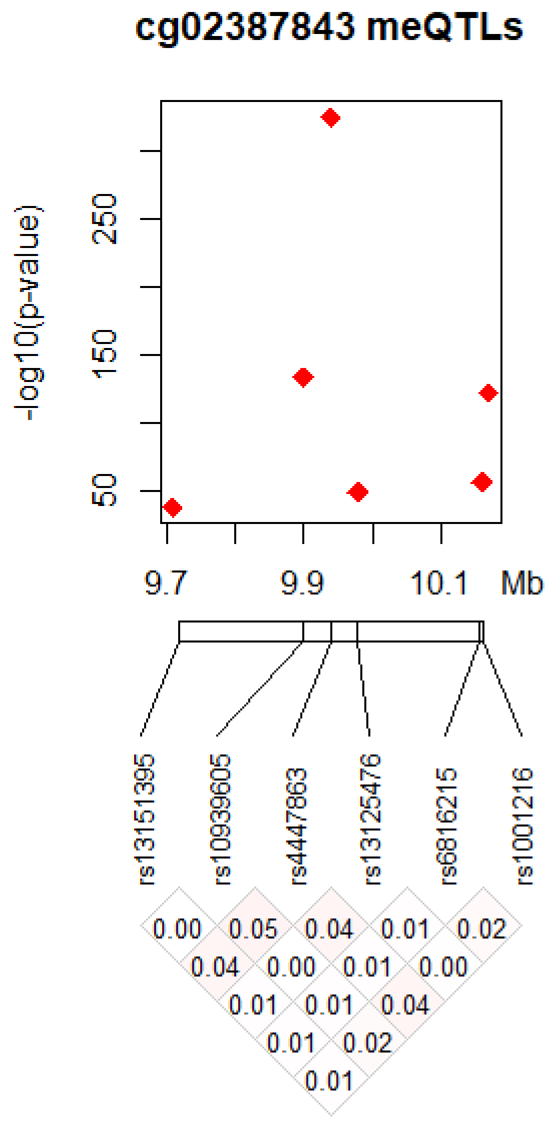

Supplementary Figure 5B

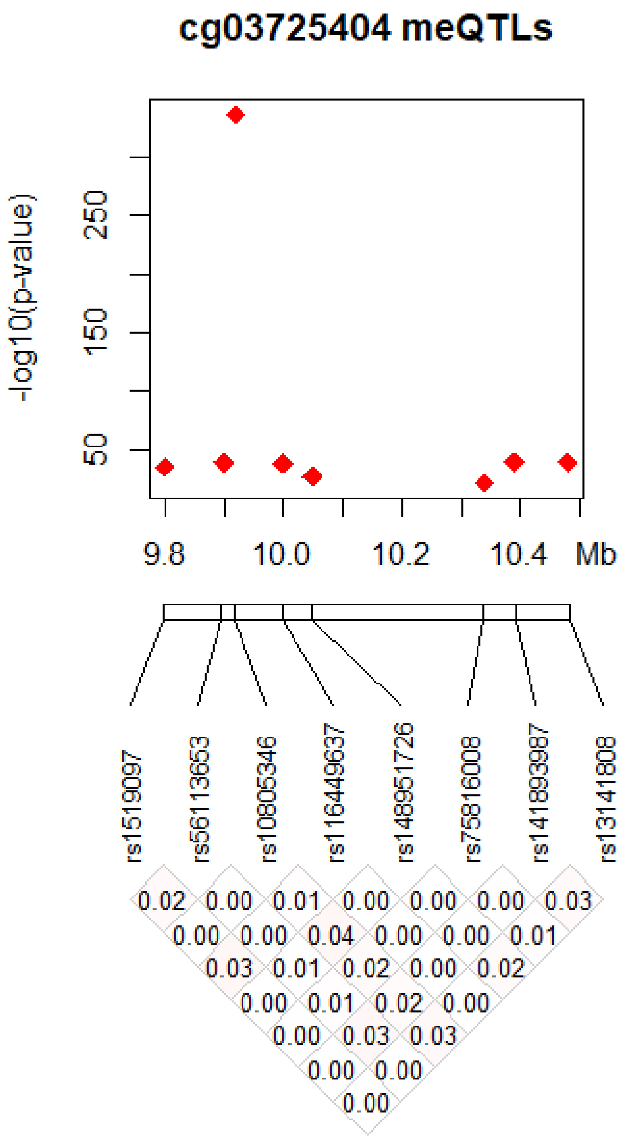

Supplementary Figure 5C

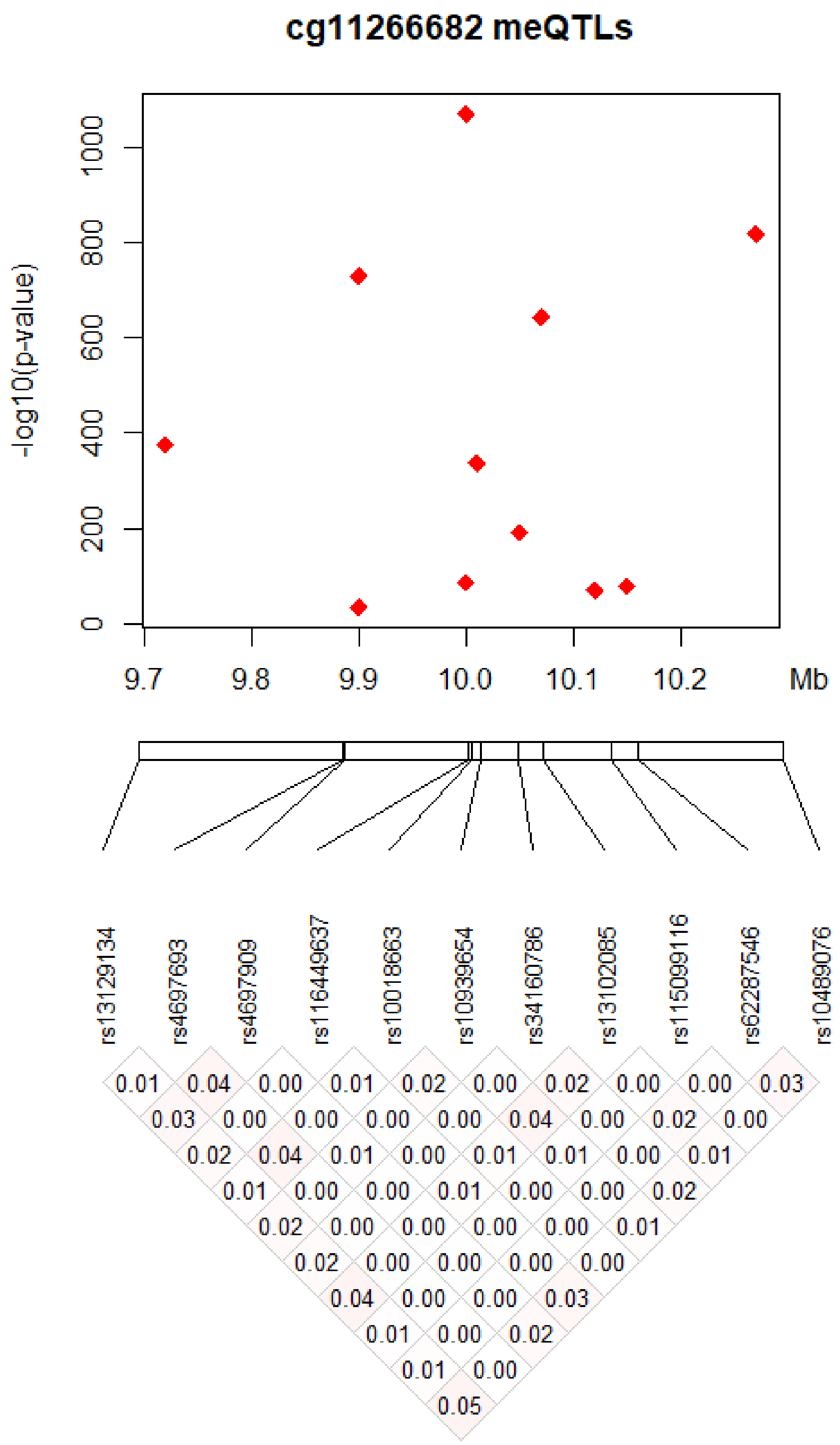

Supplementary Figure 5D

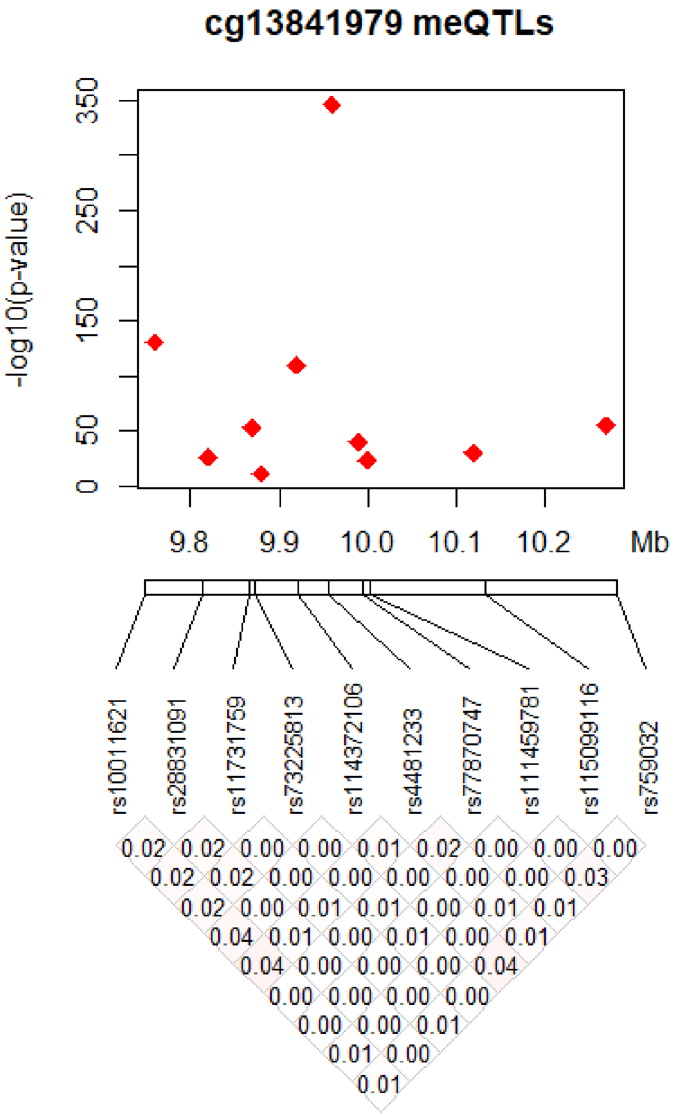

Supplementary Figure 6. For the CpG with a significant causal effect on gout, plots of the association p-value of the meQTLs (association with DNA methylation) included in the Mendelian randomization analysis (top plot) and r^2 among meQTLs (bottom plot). The p-values were 2-sided and obtained from inverse variance weighted fixed effect meta-analysis. Sample size: GoDMC meQTL study (n=27,750), r^2 (n=804).

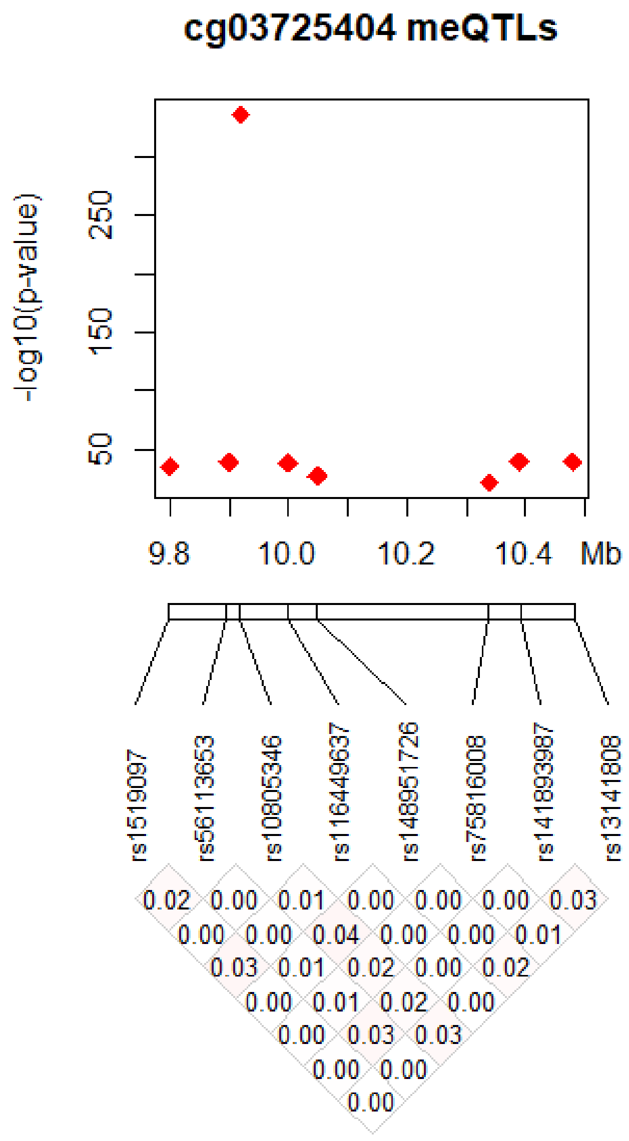

Supplementary Figure 7. Forest plot of leave-one-out analysis for the four CpGs with significant causal effect on serum urate levels: cg02387843 (A), cg03725404 (B), cg11266682 (C), and cg13841979 (D). X-axis is the causal effect estimate on serum urate in mg/dL per SD of rank-based transformed DNA methylation beta value when each of the SNPs on the y-axis was excluded as the instrument of the CpG. The whiskers represent 95% confidence intervals. Sample size: GoDMC meQTL study (n=27,750), serum urate GWAS (n=288,649). Abbreviations: MR, Mendelian randomization; SD, standard deviation.

Supplementary Figure 7A

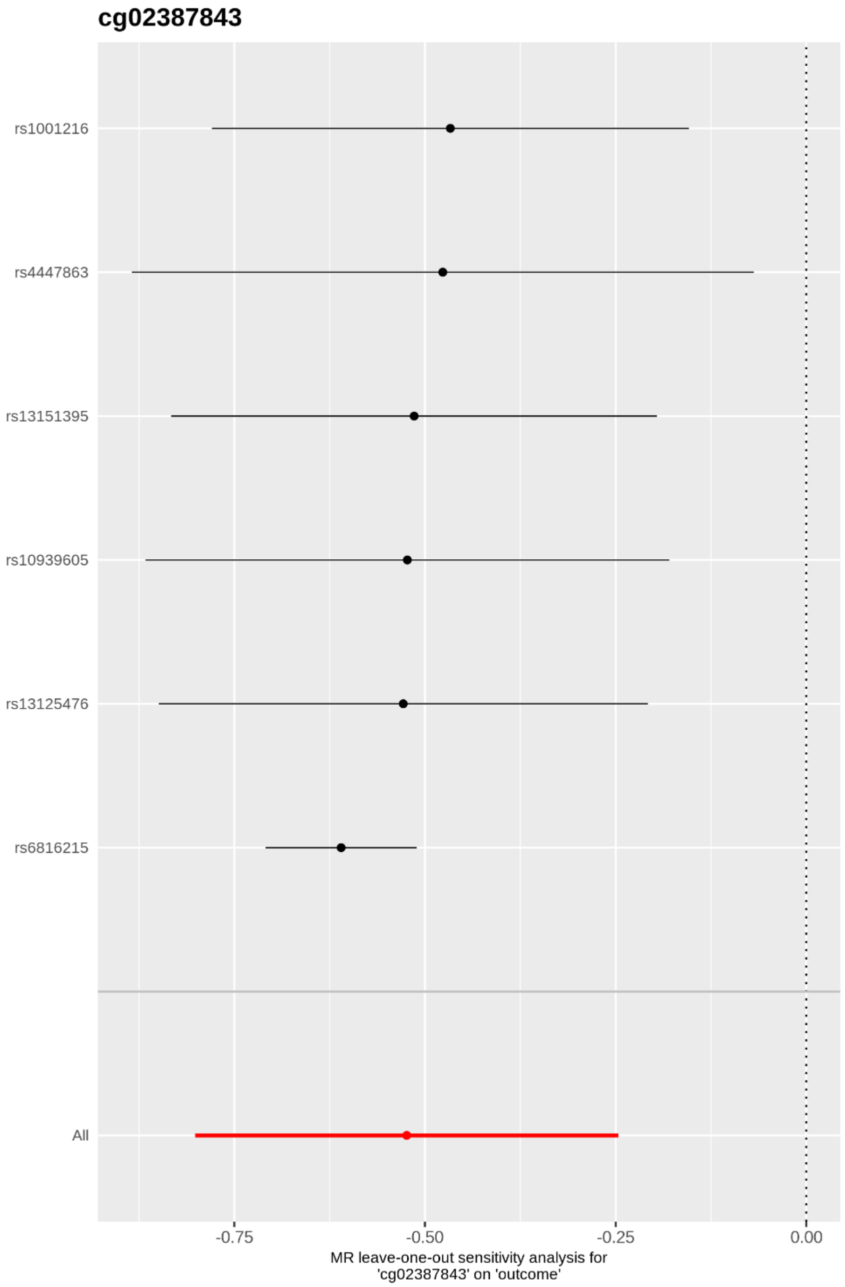

Supplementary Figure 7B

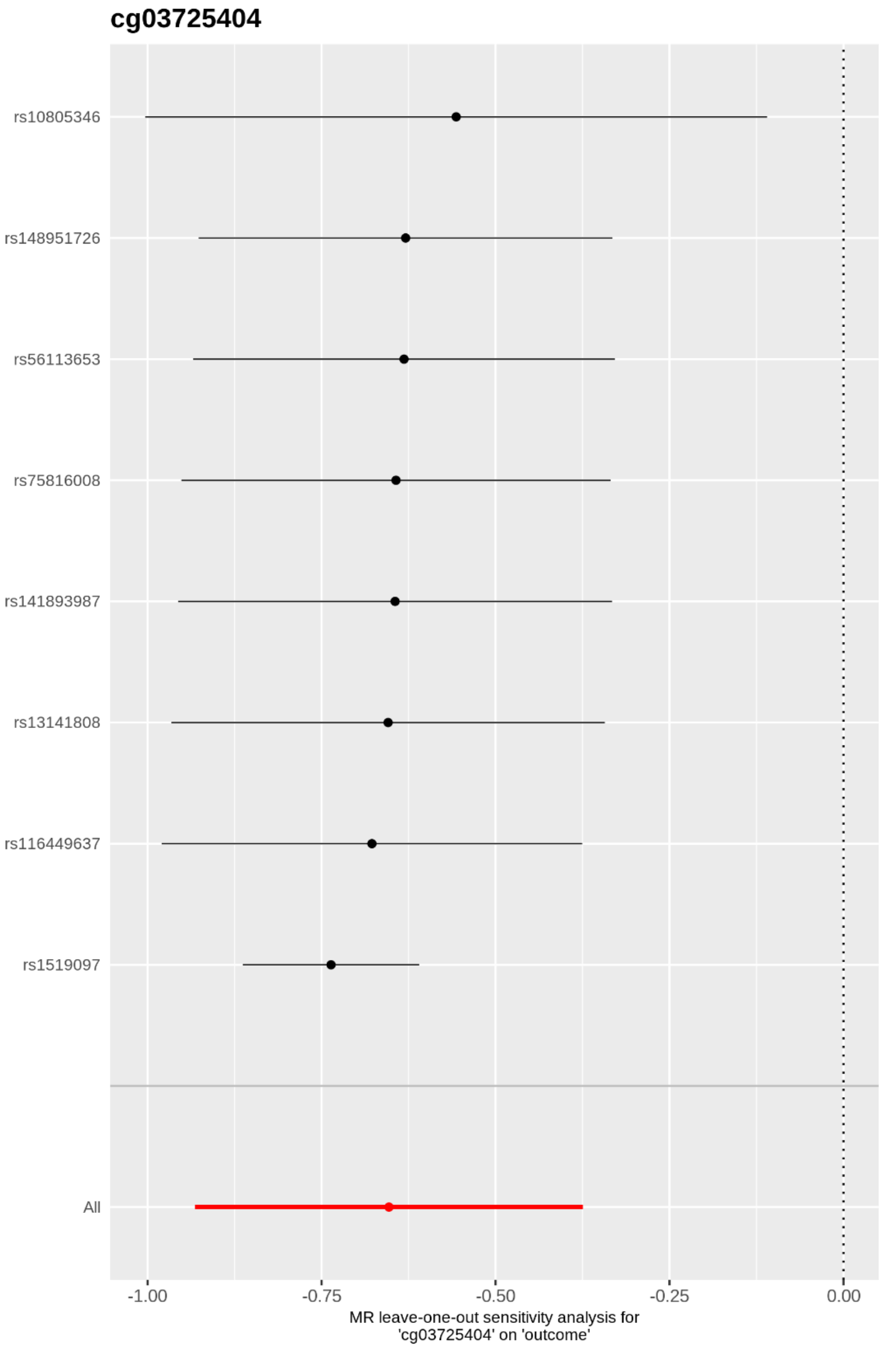

Supplementary Figure 7C

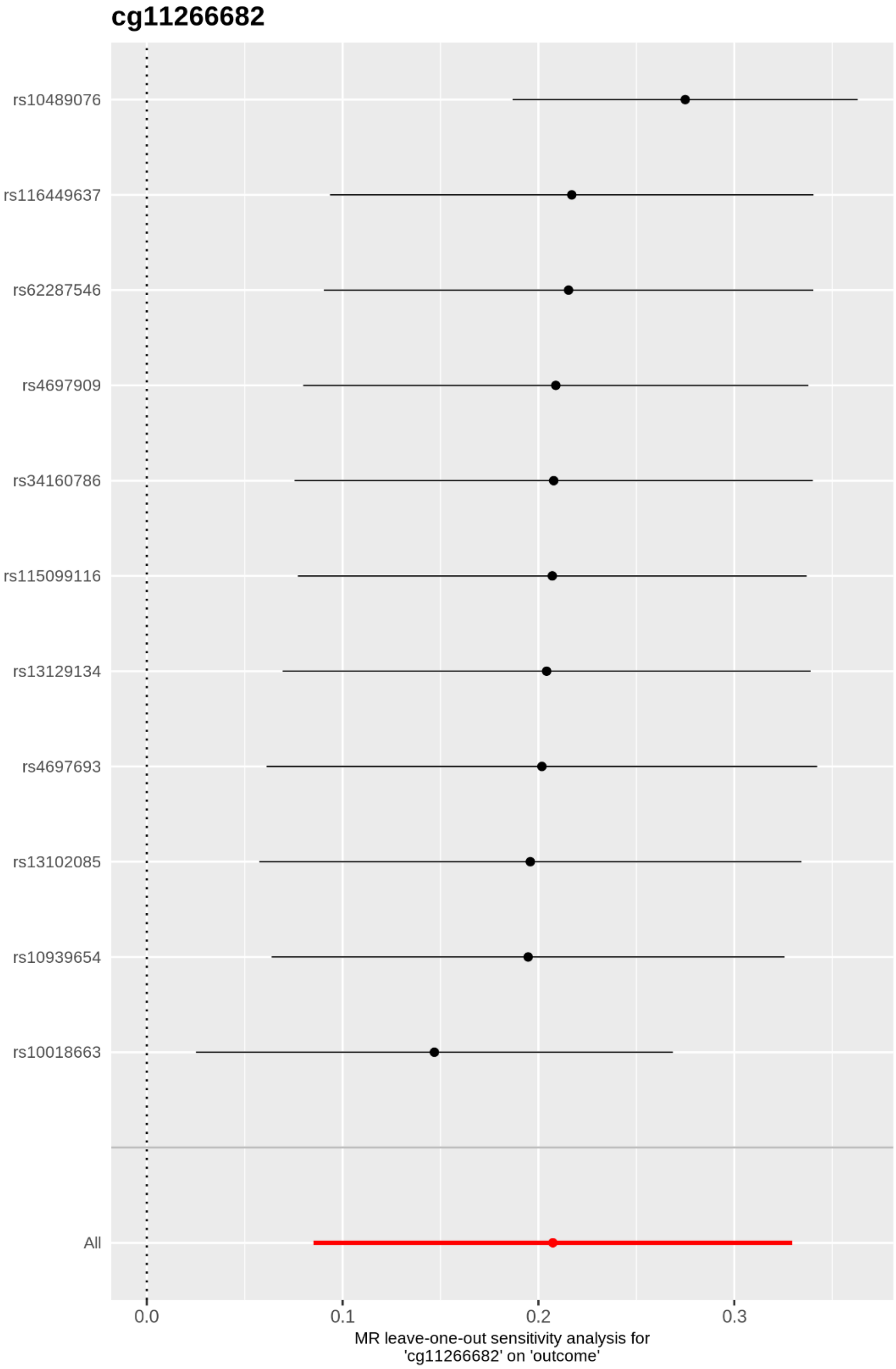

Supplementary Figure 7D

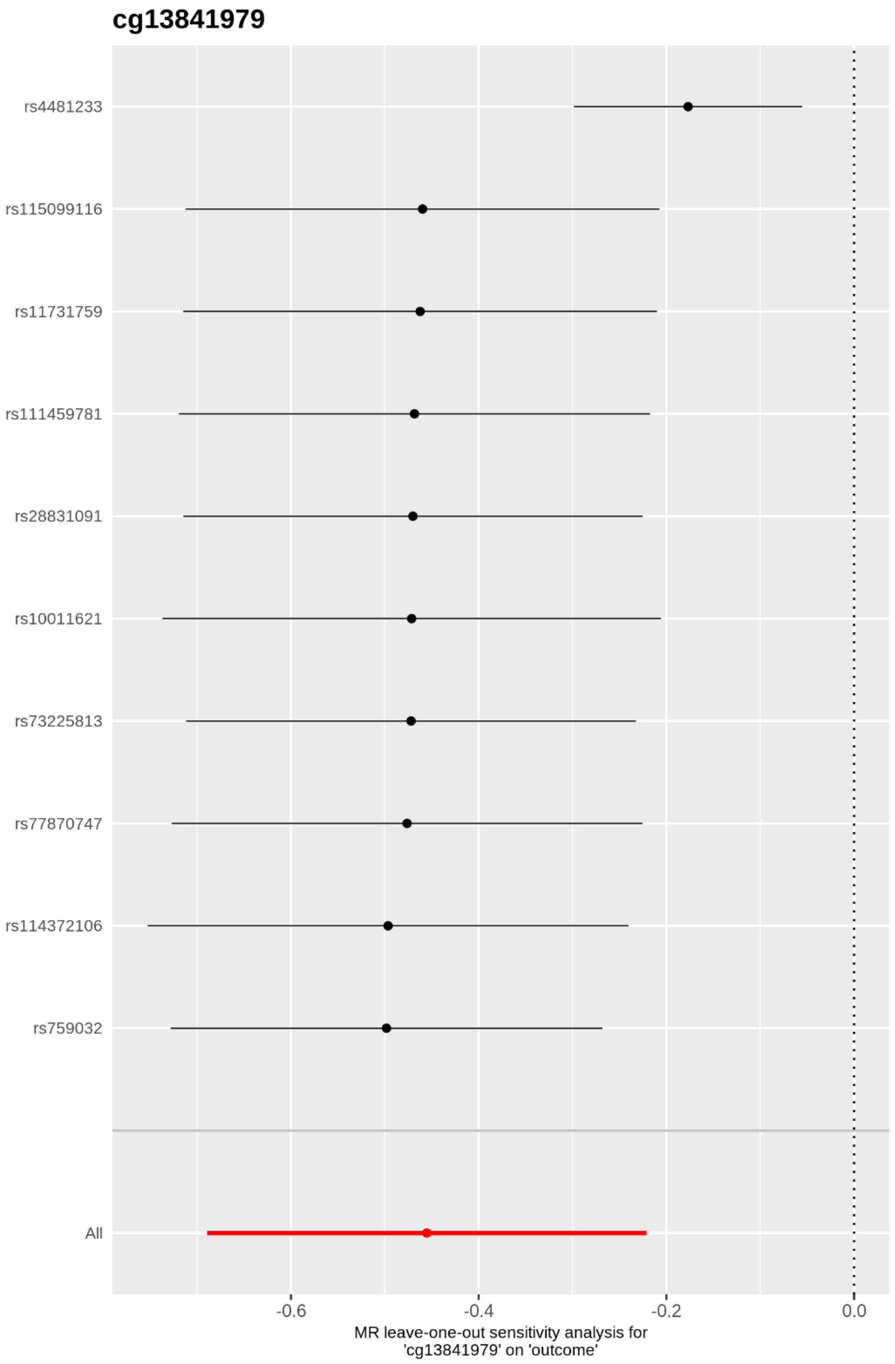

Supplementary Figure 8. Forest plot of leave-one-out analysis for the CpG with significant causal effect on gout. X-axis is the causal effect estimate on gout in log odds ratio per SD of rank-based transformed DNA methylation beta value when each of the SNPs on the y-axis was excluded as the instrument of the CpG. The whiskers represent 95% confidence intervals. Sample size: GoDMC meQTL study (n=27,750), gout GWAS (n=692,537). Abbreviations: MR, Mendelian randomization; SD, standard deviation.

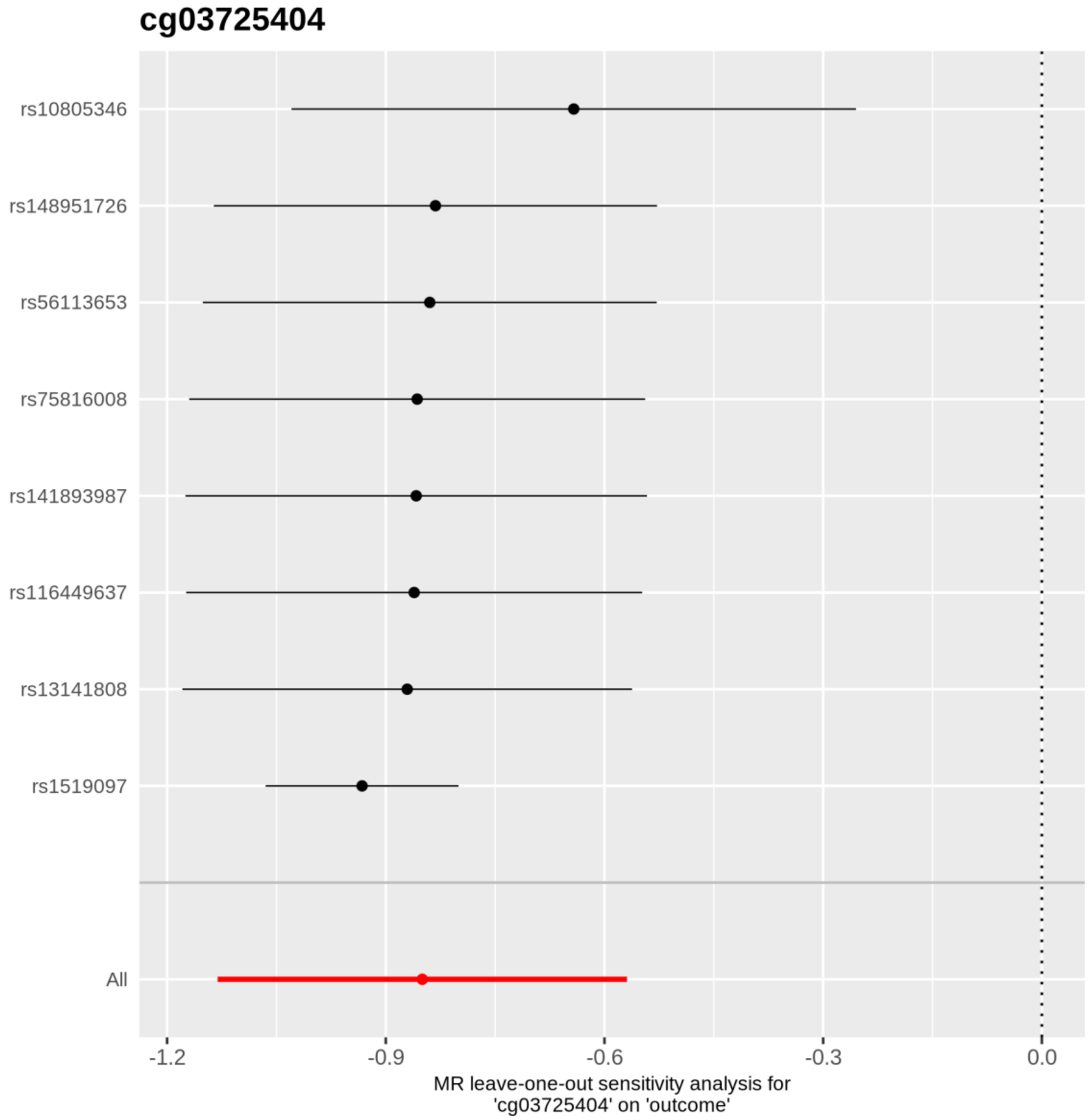

Supplementary Figure 9. Forest plot of the effects of the meQTLs on serum urate included in the forward MR analysis for the four CpGs with significant causal effects: cg02387843 (A), cg03725404 (B), cg11266682 (C), and cg13841979 (D). X-axis is the causal effect estimate on serum urate in mg/dL per SD of rank-based transformed DNA methylation beta value using each of the SNPs on the y-axis as the instrument of the CpG. The whiskers represent 95% confidence intervals. Sample size: GoDMC meQTL study (n=27,750), serum urate GWAS (n=288,649). Abbreviations: MR, Mendelian randomization; SD, standard deviation.

Supplementary Figure 9A

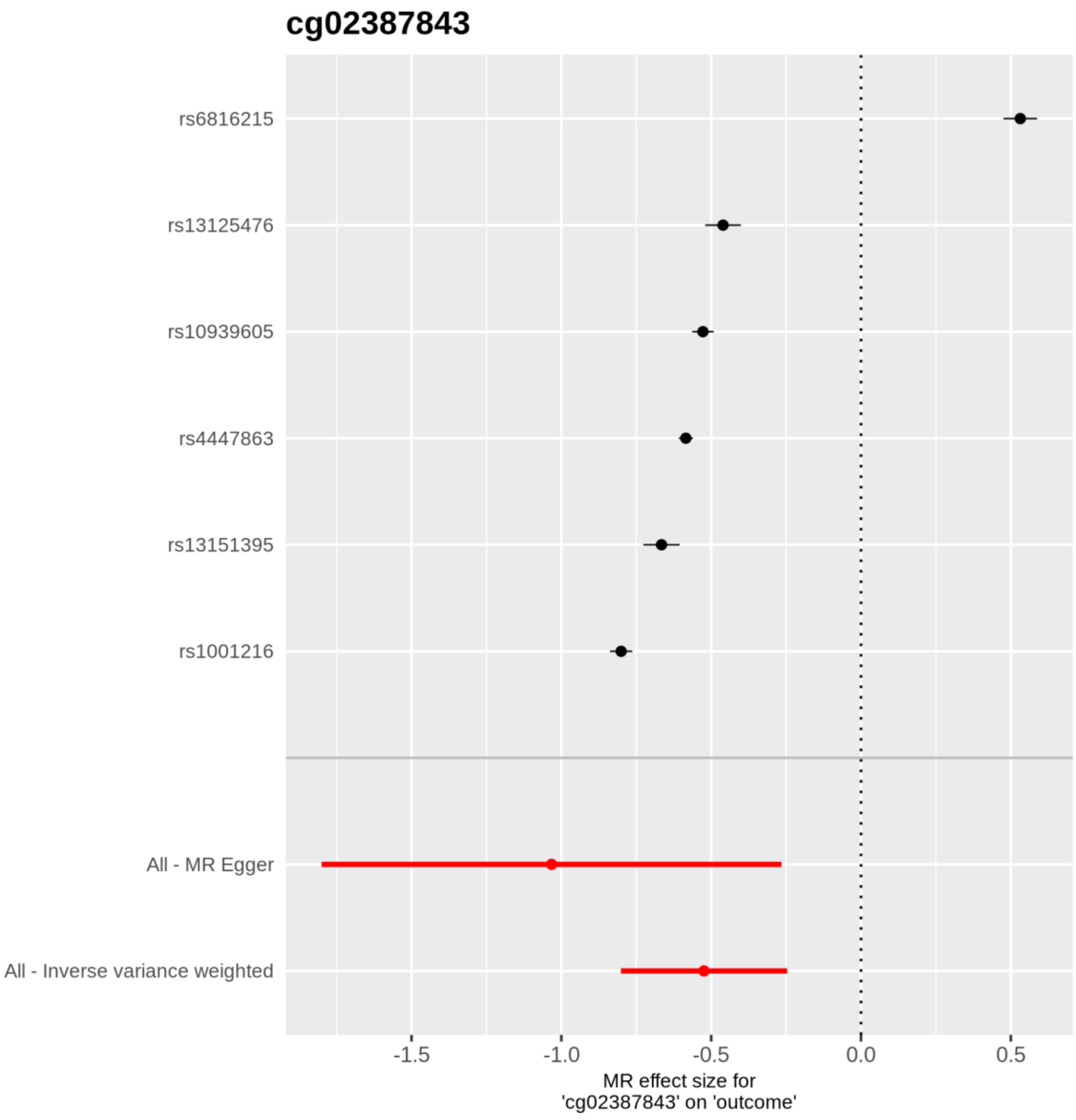

Supplementary Figure 9B

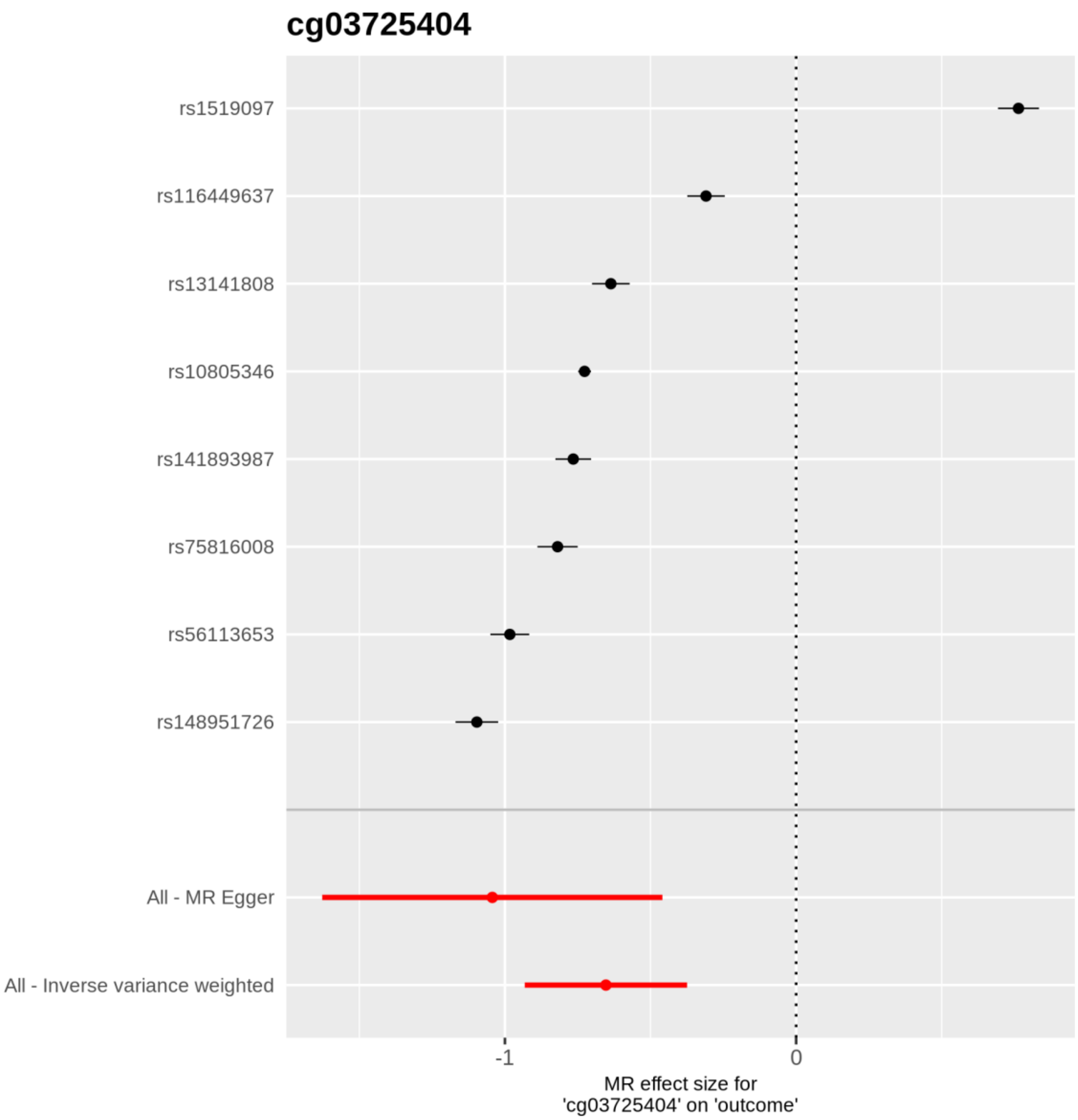

Supplementary Figure 9C

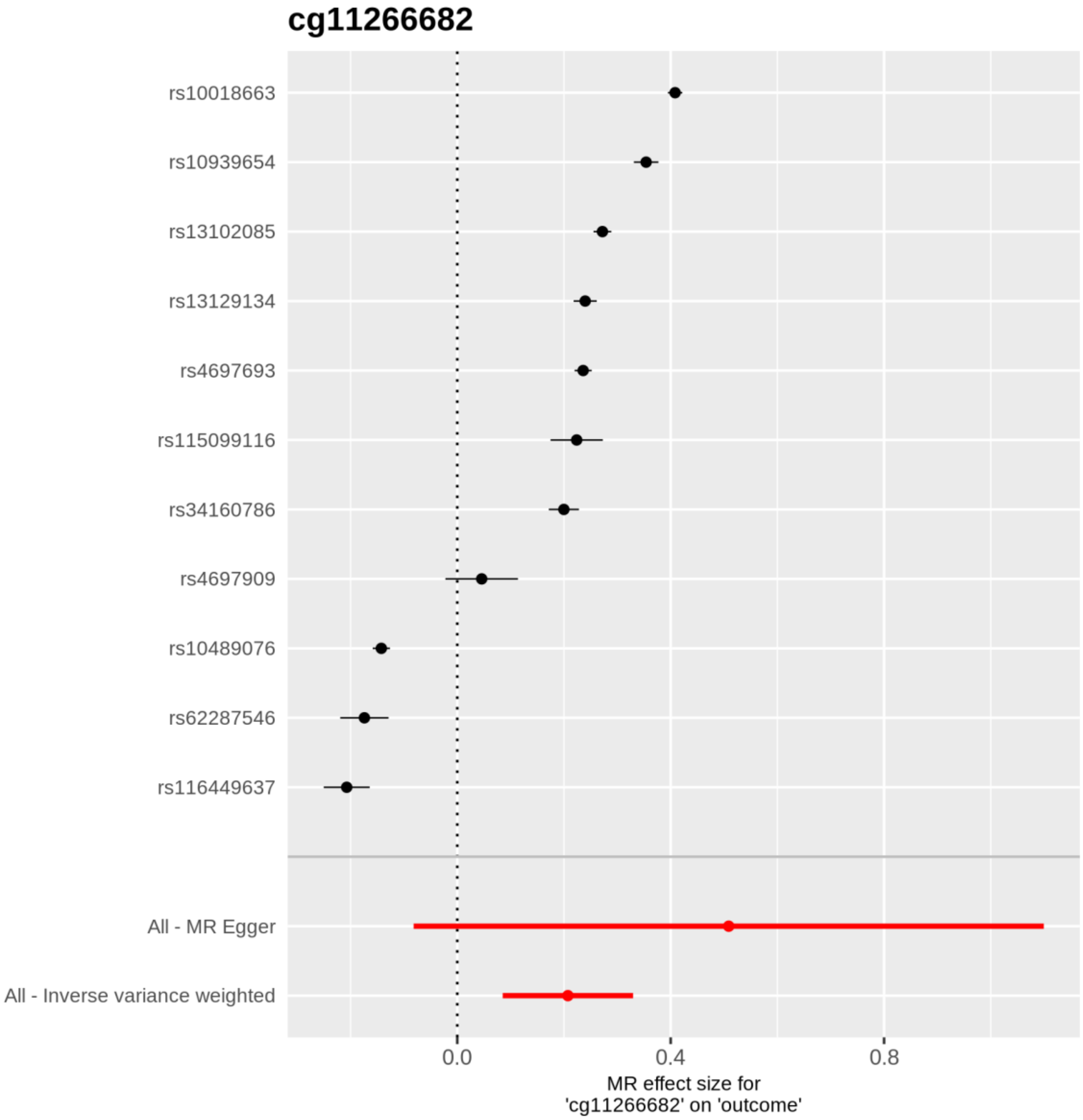

Supplementary Figure 9D

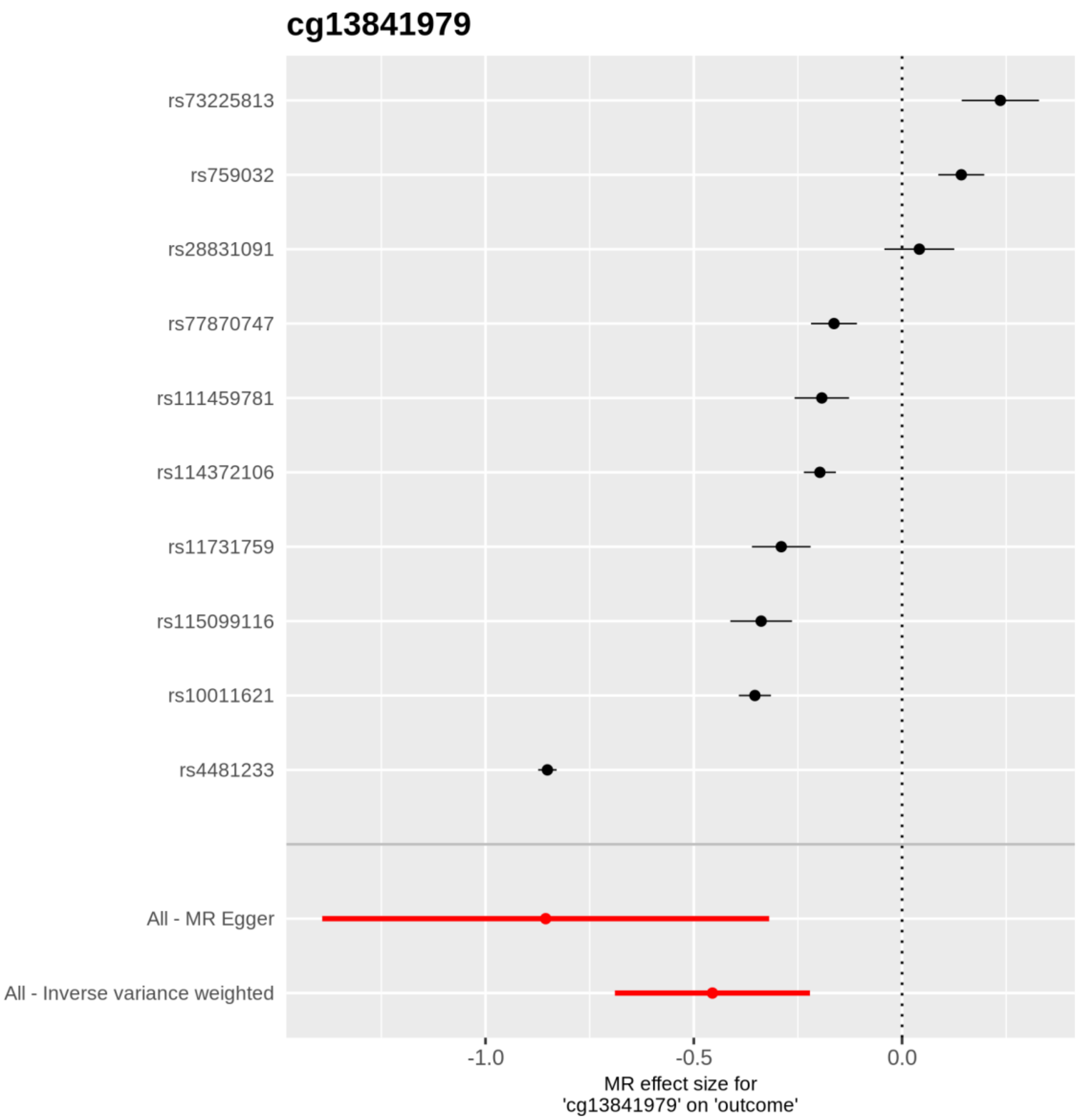

Supplementary Figure 10. Forest plot of the effects of the meQTLs on gout included in the forward MR analysis for the CpG with significant causal effects. The x-axis is the causal effect estimate on gout in log odds ratio per SD of rank-based transformed DNA methylation beta value using each of the SNPs on the y-axis as the instrument of the CpG. Sample size: GoDMC meQTL study (n=27,750), gout GWAS (n=692,537). The whiskers represent 95% confidence intervals. Abbreviations: MR, Mendelian randomization; SD, standard deviation.

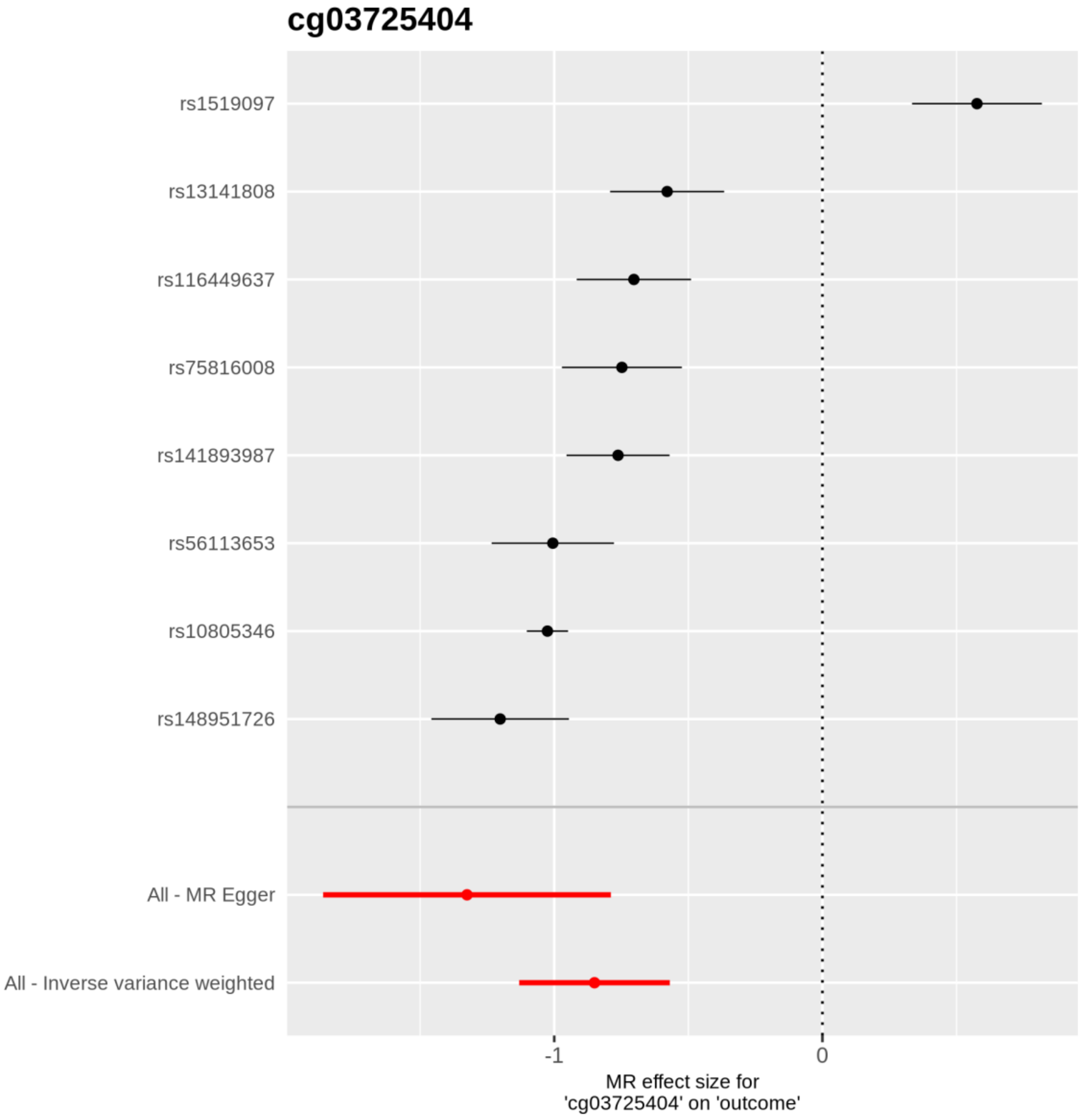

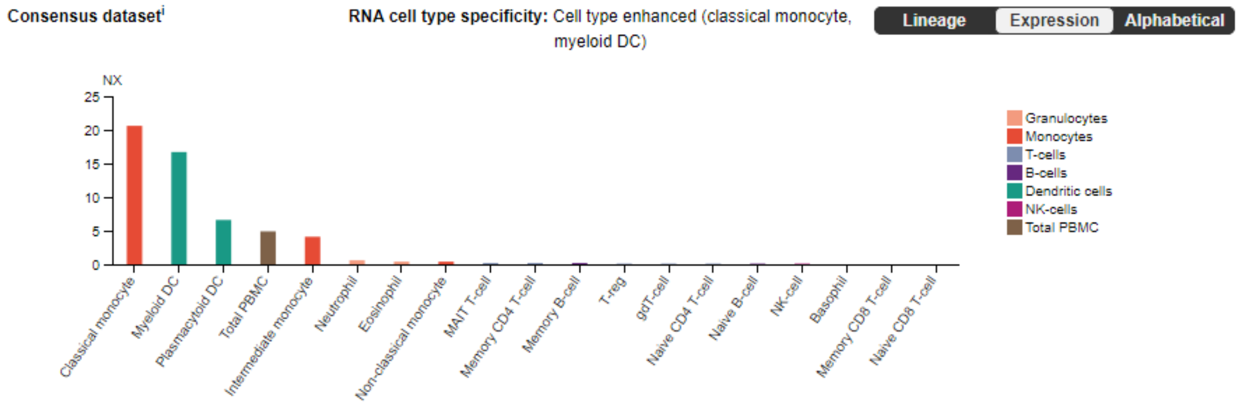

Supplementary Figure 11. *SLC2A9* gene expression among white blood cells. Abbreviations: DC, dendritic cells; PBMC, peripheral blood mononuclear cells; T-reg, regulatory T cells; NX, normalized expression. The normalized expression levels were combined from data generated by the Human Protein Atlas, GTEx and FANTOM5. The plot was downloaded from the Human Protein Atlas website on September 23, 2020.

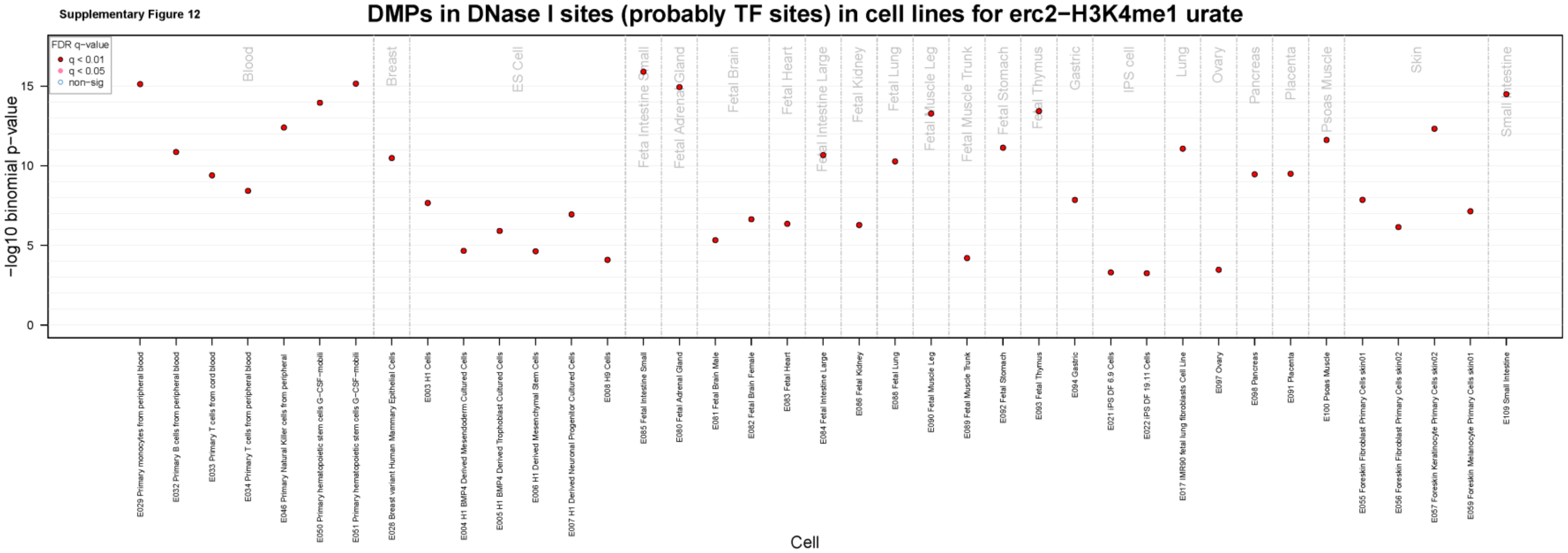

Supplementary Figure 12. Enrichment of H3K4me1 marks in the overlap between DNase I hypersensitive sites and the urate-associated CpGs (p<1E-5 in the combined meta-analysis of the discovery and replication cohorts). The enrichment p-values on the y-axis were 2-sided and obtained using binomial test. Abbreviation. DMP, differentially methylated positions of the urate-associated CpGs; TF, transcription factor; FDR, false discovery rate using the Benjamini-Yekutieli method.

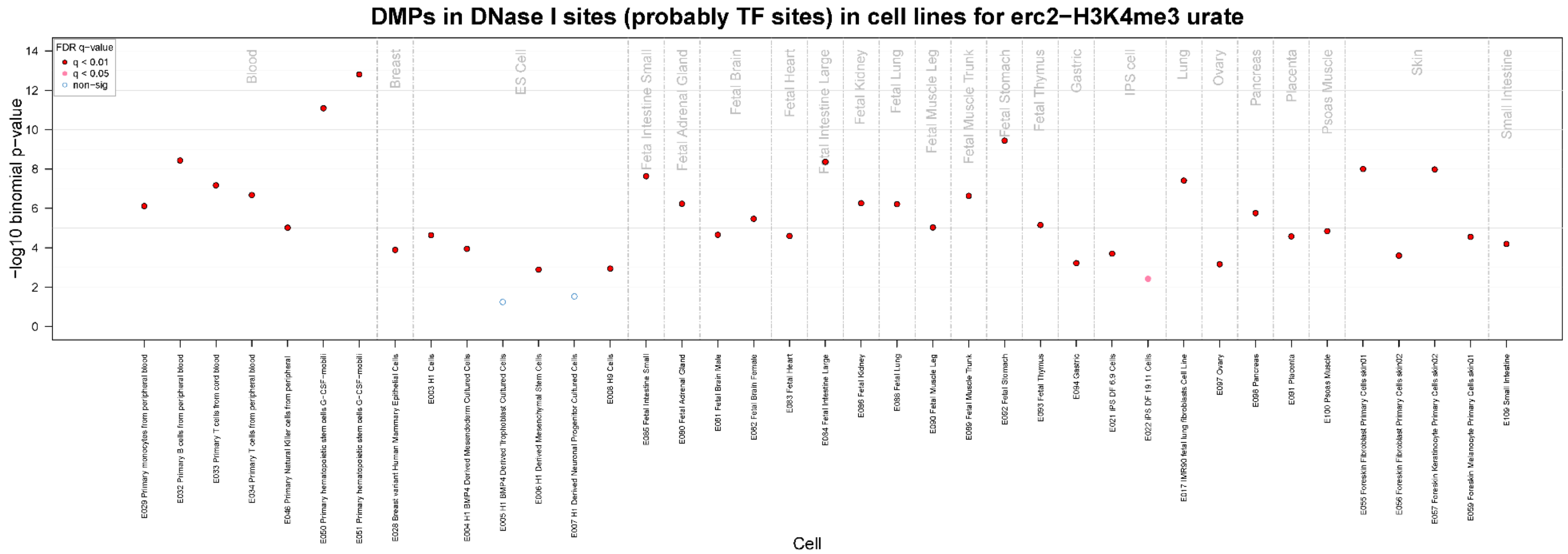

Supplementary Figure 13. Enrichment of H3K4me3 marks in the overlap between DNase I hypersensitive sites and the urate-associated CpGs (p<1E-5 in the combined meta-analysis of the discovery and replication cohorts). The enrichment p-values on the y-axis were 2-sided and obtained using binomial test. Abbreviation. DMP, differentially methylated positions of the urate-associated CpGs; TF, transcription factor; FDR, false discovery rate using the Benjamini-Yekutieli method.

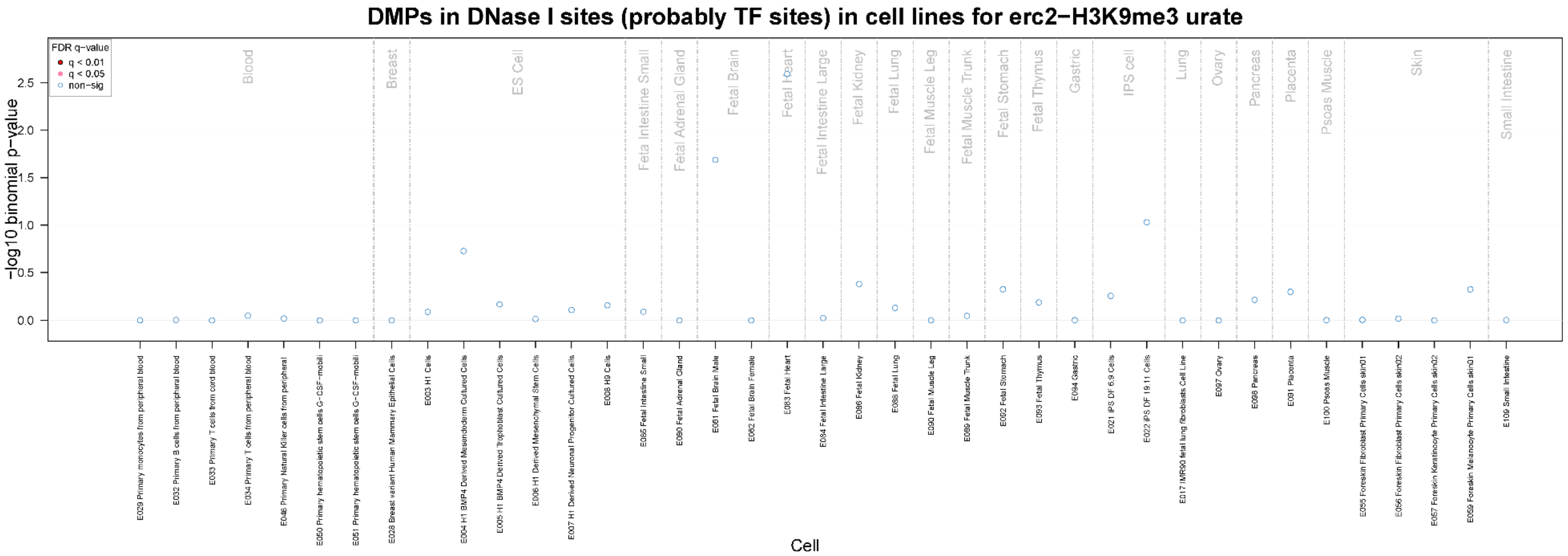

Supplementary Figure 14. Enrichment of H3K9me3 marks in the overlap between DNase I hypersensitive sites and the urate-associated CpGs (p<1E-5 in the combined meta-analysis of the discovery and replication cohorts). The enrichment p-values on the y-axis were 2-sided and obtained using binomial test. Abbreviation. DMP, differentially methylated positions of the urate-associated CpGs; TF, transcription factor; FDR, false discovery rate using the Benjamini-Yekutieli method.

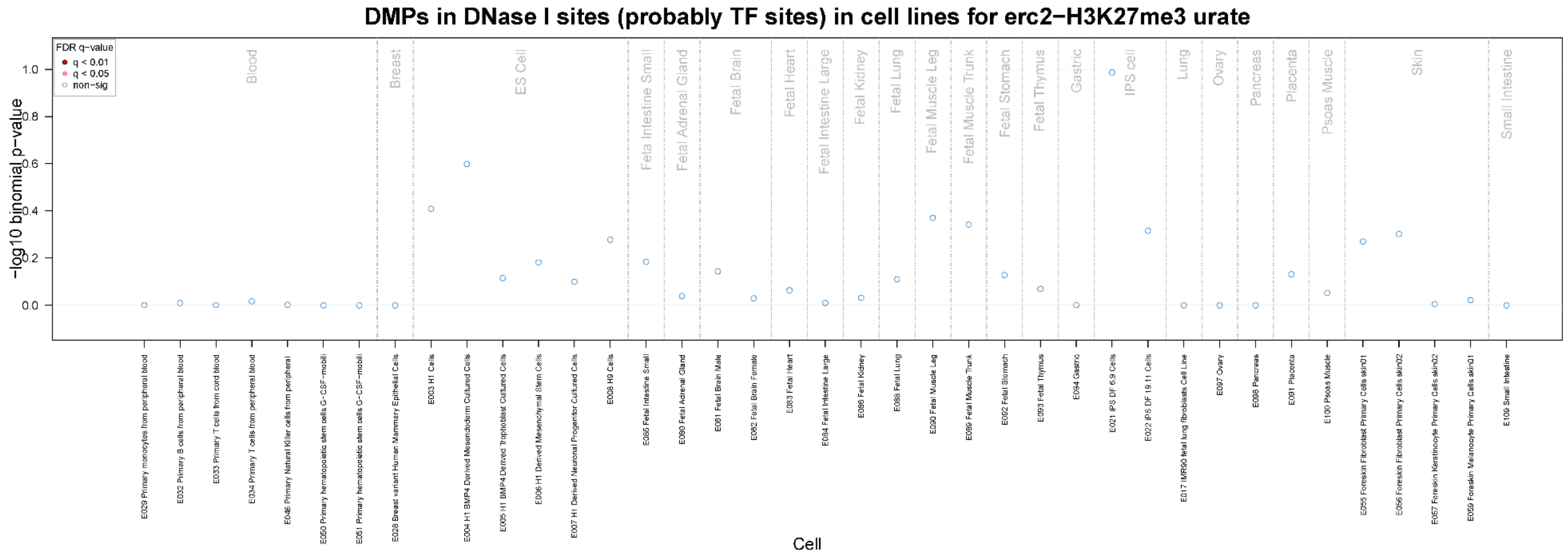

Supplementary Figure 15. Enrichment of H3K27me3 marks in the overlap between DNase I hypersensitive sites and the urate-associated CpGs (p<1E-5 in the combined meta-analysis of the discovery and replication cohorts). The enrichment p-values on the y-axis were 2-sided and obtained using binomial test. Abbreviation. DMP, differentially methylated positions of the urate-associated CpGs; TF, transcription factor; FDR, false discovery rate using the Benjamini-Yekutieli method.

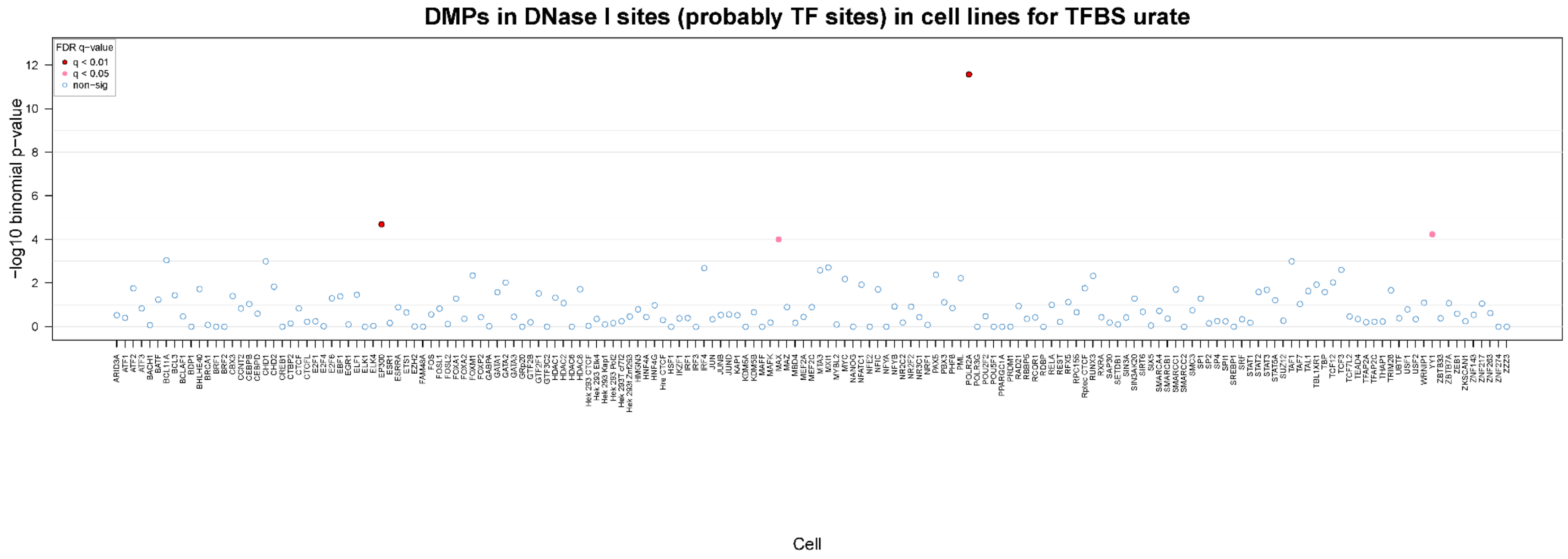

Supplementary Figure 16. Enrichment of transcription factor binding sites in the overlap between DNase I hypersensitive sites and the urate-associated CpGs (p<1E-5 in the combined meta-analysis of the discovery and replication cohorts). The enrichment p-values on the y-axis were 2-sided and obtained using binomial test. Abbreviation. DMP, differentially methylated positions of the urate-associated CpGs; TF, transcription factor; FDR, false discovery rate using the Benjamini-Yekutieli method.

Supplementary Figure 17. Forest plot of leave-one-out analysis showing that the causal estimates of serum urate on 5 CpGs were driven by the urate GWAS index SNP at *SLC2A9,* rs4447862, and became insignificant after removing rs4447862: cg01881899 (A), cg02387843 (B), cg14348967 (C), cg18125510 (D), and cg22821355 (E). The x-axis is the causal effect estimate in SD of rank-based transformed DNA methylation beta value per mg/dL of serum urate using each of the SNPs on the y-axis as the instrument for serum urate. Sample size: serum urate GWAS (n=288,649), meQTL from FHS (n=3,866). The whiskers represent 95% confidence interval. Abbreviation: MR, Mendelian randomization; SD, standard deviation.

Supplementary Figure 17A

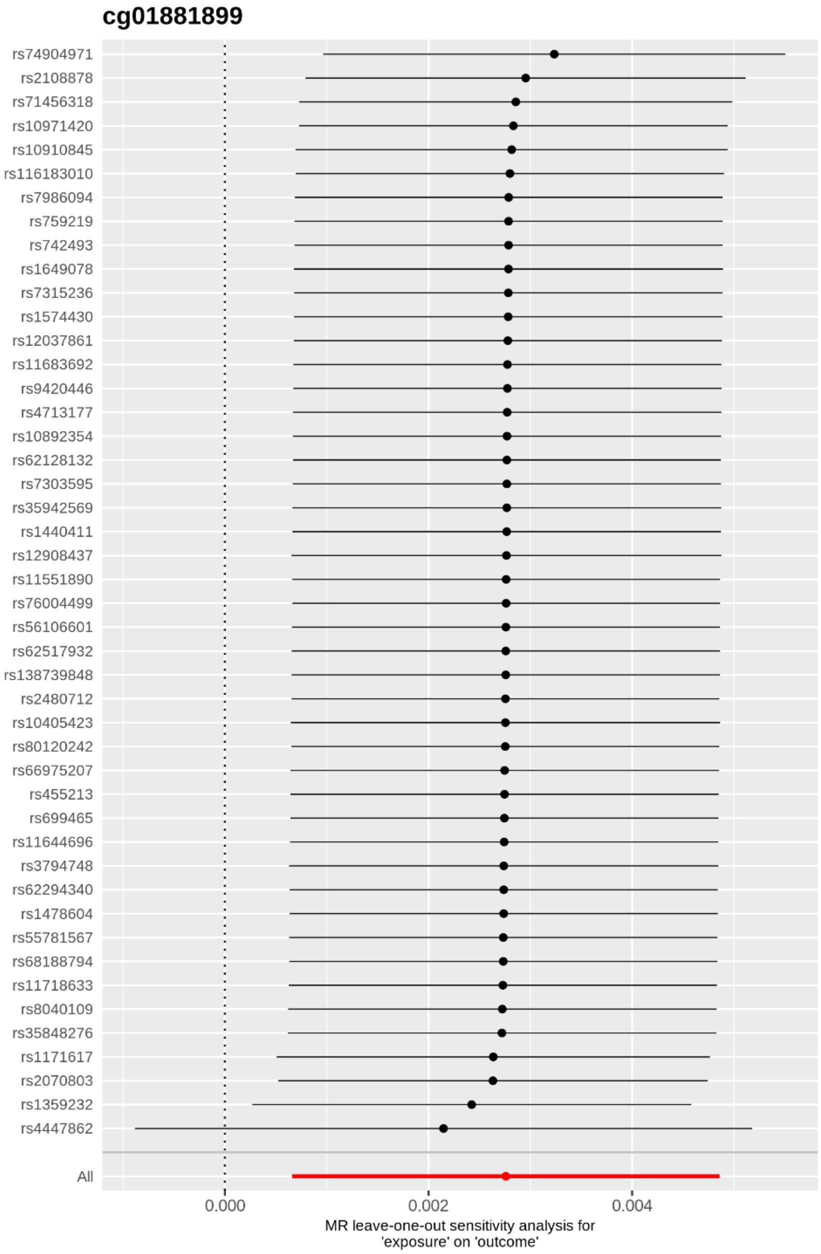

Supplementary Figure 17B

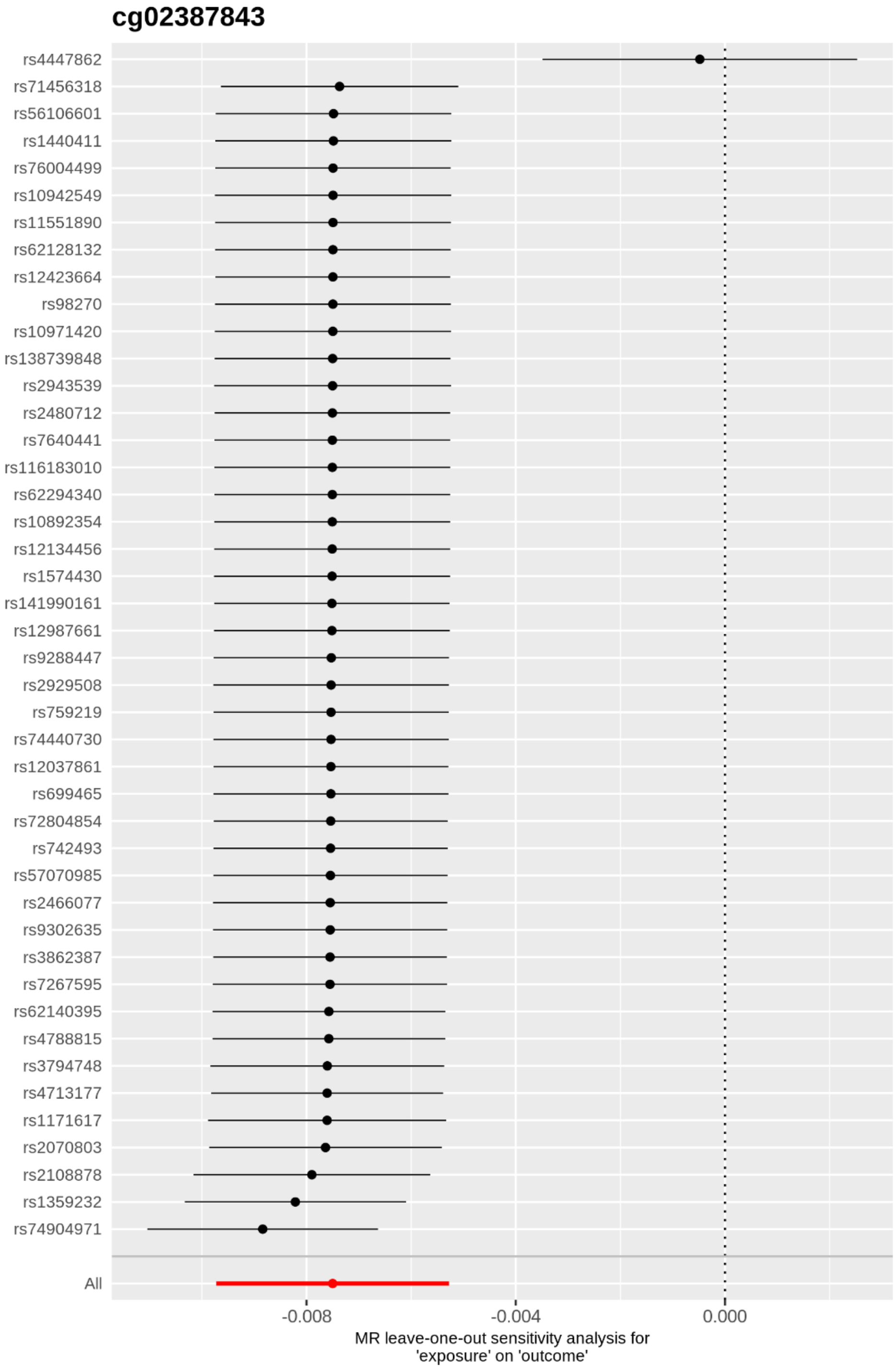

Supplementary Figure 17C

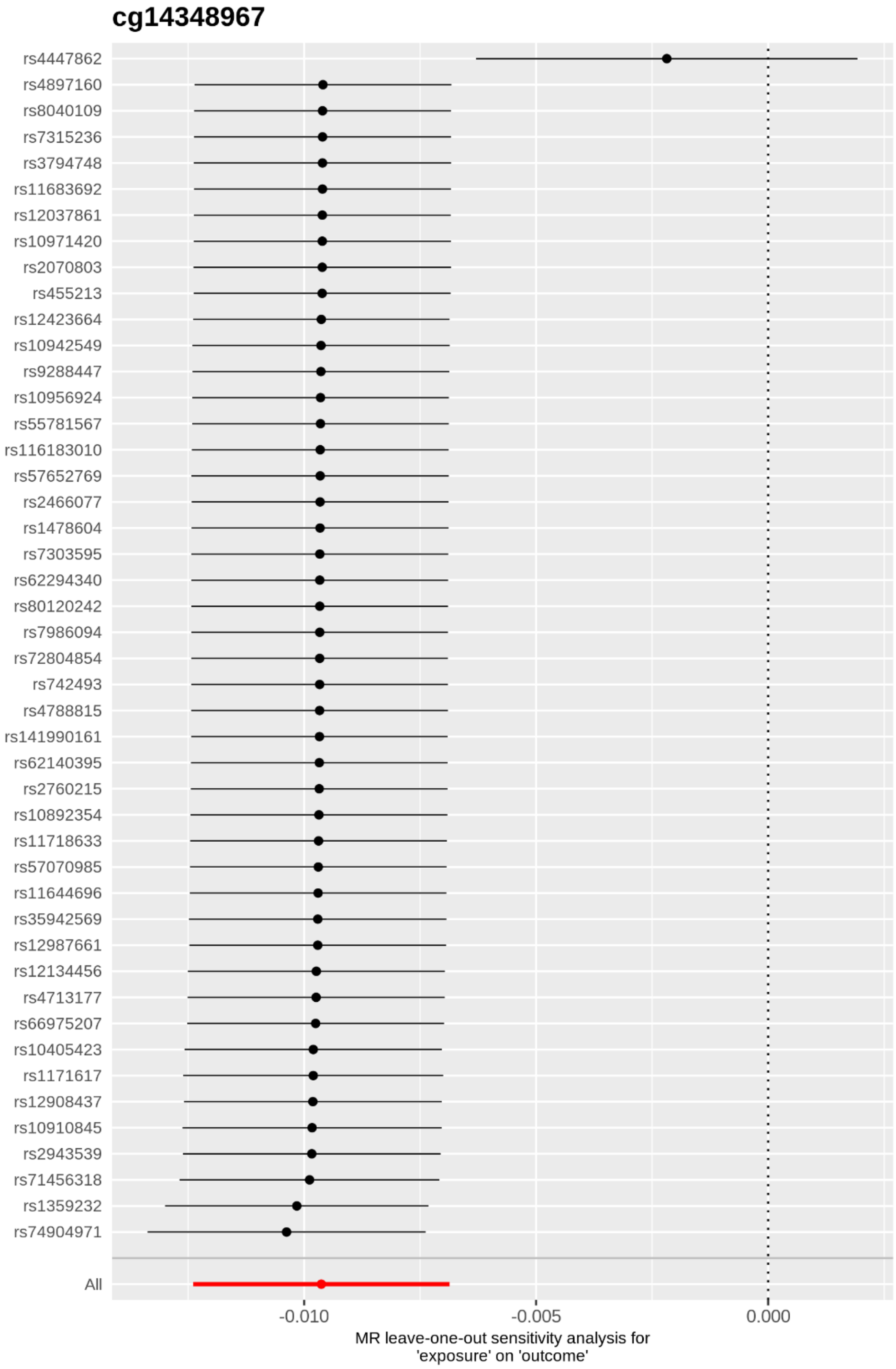

Supplementary Figure 17D

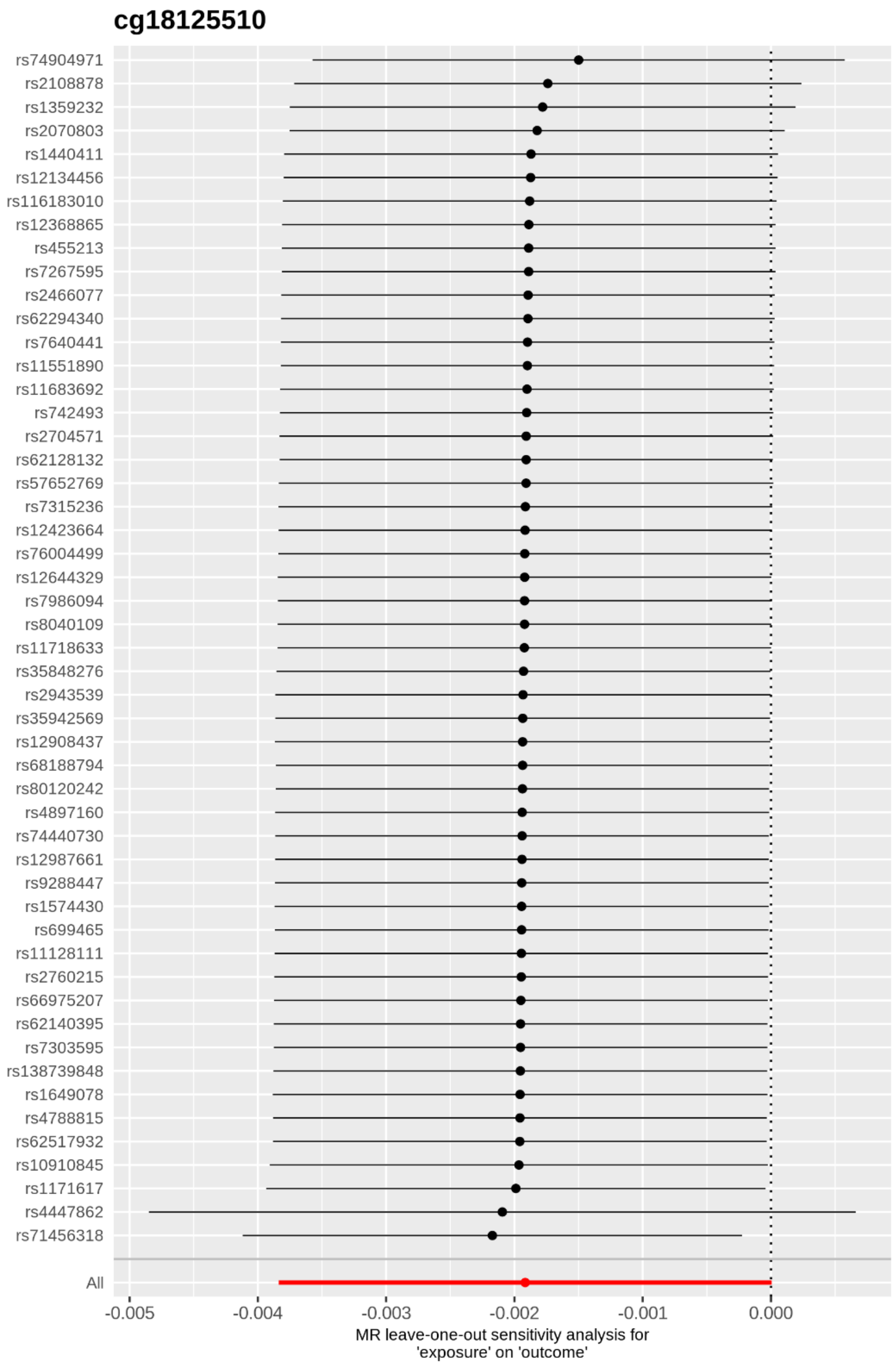

Supplementary Figure 17E

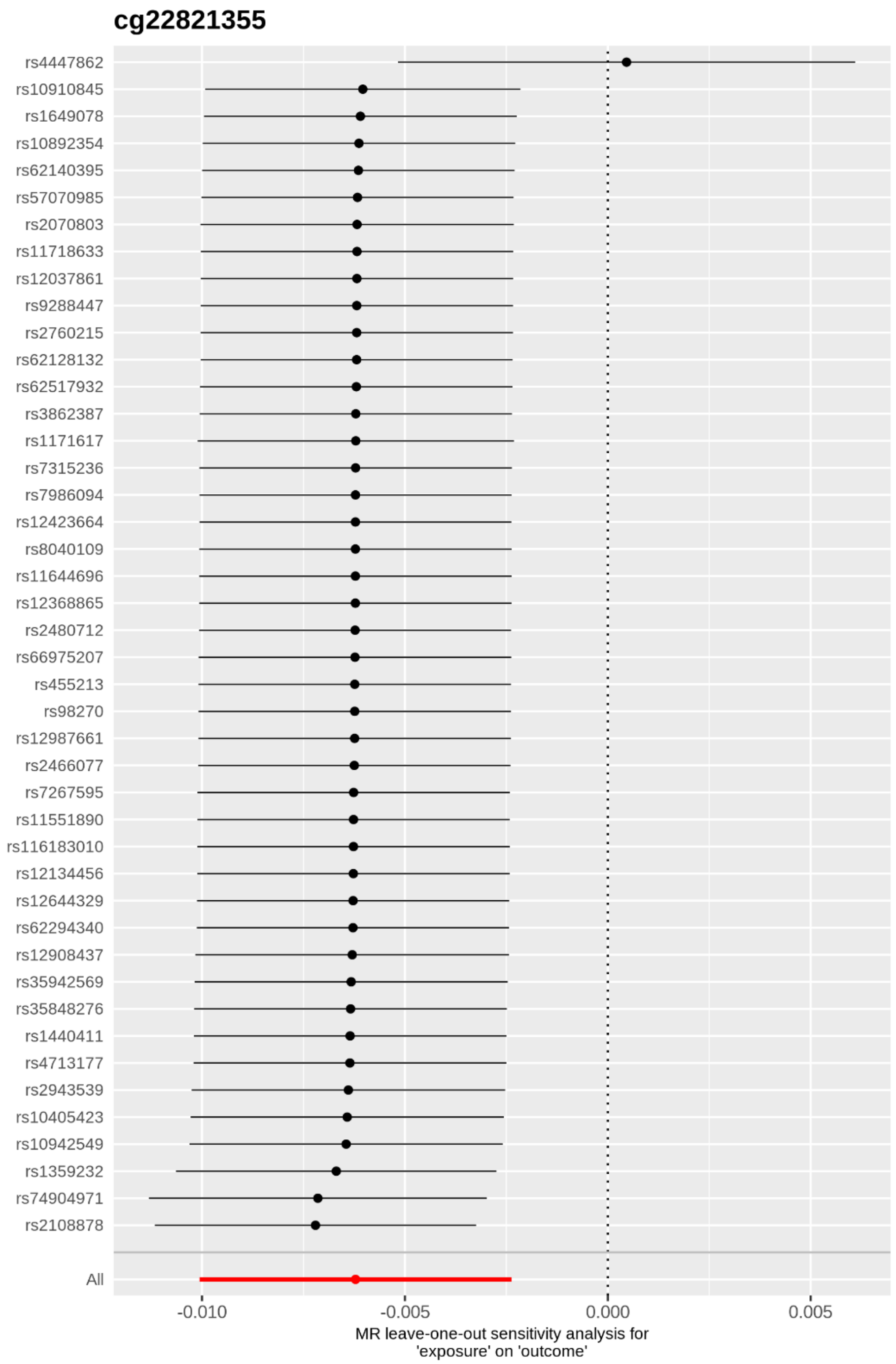

Supplement: Supplementary file 1 — Supplementary Information [file 41467_2021_27198_MOESM1_ESM.pdf]
