# Supplementary material for: Epigenome-wide association study of serum urate reveals insights into urate co-regulation and the SLC2A9 locus
Source: Nat Commun. 2021 Dec 9;12:7173. doi: 10.1038/s41467-021-27198-4 (PMC8660809; doi:10.1038/s41467-021-27198-4)
Supplement: Supplementary file 3 — Description of Additional Supplementary Files [file 41467_2021_27198_MOESM3_ESM.pdf]

Description of Additional Supplementary Files

File Name: Supplementary Data 1
Description: Population characteristics of participating cohorts

File Name: Supplementary Data 2
Description: Cohort-specific methods

File Name: Supplementary Data 3
Description: Measured or estimated white blood cell proportions used in analysis among participating cohorts, mean (SD) in %

File Name: Supplementary Data 4
Description: Replicated CpGs from meta-analysis of epigenome-wide association studies of serum urate

File Name: Supplementary Data 5
Description: CpGs with p-value < 1.1E-7 in the meta-analysis of 17 cohorts of European ancestry

File Name: Supplementary Data 6
Description: CpGs with p-value < 1.1E-7 in the meta-analysis of five cohorts of African-American ancestry

File Name: Supplementary Data 7
Description: CpGs with p-value < 1.1E-7 in the cohort of participants of South Asian ancestry (LOLIPOP)

File Name: Supplementary Data 8
Description: CpGs with p-value < 1E-5 in the cohort of Sub-Saharan Africans (RODAM)

File Name: Supplementary Data 9
Description: Heritability estimates of the replicated sites of serum urate

File Name: Supplementary Data 10
Description: Results of sensitivity analysis of the causal effects of CpGs on serum urate

File Name: Supplementary Data 11
Description: Results of sensitivity analysis of the causal effects of urate-associated CpGs on gout

File Name: Supplementary Data 12
Description: CpG mediating effect for 4 independent SNPs at SLC2A9 in the ARIC European ancestry cohort.

File Name: Supplementary Data 13
Description: CpG mediating effects for two independent GWAS SNPs of serum urate at SLC2A9 from a meta-analysis of three cohorts

File Name: Supplementary Data 14
Description: Association between replicated CpGs and gene expression in monocytes (Kennedy et al. BMC Genomics 2018). The summary statistics of the gene expression association were available only for those with gene expression association p-value <1e-5.

File Name: Supplementary Data 15
Description: Association between the urate-associated CpGs and gene expression in whole blood

File Name: Supplementary Data 16
Description: Association between urate-associated CpGs at SLC2A9 and gene expression in tubulointerstitial kidney tissue

File Name: Supplementary Data 17
Description: Nearest genes of urate-associated CpGs with differential gene expression in kidney and intestine tissues of Abcg2 Q140K male knock-out mice versus wild type

File Name: Supplementary Data 18
Description: List of significant GO terms and KEGG and Reactome pathways from enrichment analysis

File Name: Supplementary Data 19
Description: Publications of EWAS cardiometabolic traits that were included in the lookup for the urate-associated CpGs

File Name: Supplementary Data 20
Description: Significant CpGs in the lookup from EWAS of cardiometabolic traits

File Name: Supplementary Data 21
Description: Association of urate-associated CpGs that were also reported to be significant in EWAS of metabolic traits quantified with nuclear magnetic resonance (Gomez-Alonso et al. Clinical Epigenetics 2021)

File Name: Supplementary Data 22
Description: Source of GWAS summary statistics of potential confounders used in forward and reverse Mendelian randomization analyses
