# Supplementary material for: Epigenome-wide association study of serum urate reveals insights into urate co-regulation and the SLC2A9 locus
Source: Nat Commun. 2021 Dec 9;12:7173. doi: 10.1038/s41467-021-27198-4 (PMC8660809; doi:10.1038/s41467-021-27198-4)
Supplement: Supplementary file 5 — Reporting Summary [file 41467_2021_27198_MOESM5_ESM.pdf]

nature portfolio

Corresponding author(s): Adrienne Tin, Anna Kottgen

Last updated by author(s): Oct 25, 2021

Reporting Summary

Nature Portfolio wishes to improve the reproducibility of the work that we publish. This form provides structure for consistency and transparency in reporting. For further information on Nature Portfolio policies, see our Editorial Policies and the Editorial Policy Checklist.

Statistics

For all statistical analyses, confirm that the following items are present in the figure legend, table legend, main text, or Methods section.

n/a	Confirmed	
☐	☒	The exact sample size (*n*) for each experimental group/condition, given as a discrete number and unit of measurement
☐	☒	A statement on whether measurements were taken from distinct samples or whether the same sample was measured repeatedly
☐	☒	The statistical test(s) used AND whether they are one- or two-sided<br>*Only common tests should be described solely by name; describe more complex techniques in the Methods section.*
☐	☒	A description of all covariates tested
☐	☒	A description of any assumptions or corrections, such as tests of normality and adjustment for multiple comparisons
☐	☒	A full description of the statistical parameters including central tendency (e.g. means) or other basic estimates (e.g. regression coefficient) AND variation (e.g. standard deviation) or associated estimates of uncertainty (e.g. confidence intervals)
☐	☒	For null hypothesis testing, the test statistic (e.g. *F*, *t*, *r*) with confidence intervals, effect sizes, degrees of freedom and *P* value noted<br>*Give P values as exact values whenever suitable.*
☐	☒	For Bayesian analysis, information on the choice of priors and Markov chain Monte Carlo settings
☐	☒	For hierarchical and complex designs, identification of the appropriate level for tests and full reporting of outcomes
☐	☒	Estimates of effect sizes (e.g. Cohen's *d*, Pearson's *r*), indicating how they were calculated

Our web collection on statistics for biologists contains articles on many of the points above.

Software and code

Policy information about availability of computer code

Data collection	No software was used for data collection.
Data analysis	- QC-tools for EWAS summary statistics: R-based in-house pipeline, R-packages: metafor (v2.4.0), qqman (v0.1.4), limma (v3.42.2), openxlsx (v4.1.5), car (v3.0.8)<br>-Software-tools for association analyses: R-based in-house pipeline, R-packages: metafor (v2.4.0), qqman (v0.1.4), limma (v3.42.2), bacon (v1.16.0), mutoss (v0.1.12)<br>- Software-tools for postprocessing of results: R-based in-house pipeline, R-packages: metafor (v2.4.0), methylGSA (v1.6.1), ggplot2 (v3.3.3), rmeta (v3.0), SeSAMe v1.10.5<br>-mediation R package v4.5.0<br>-TwoSampleMR R package v0.5.6<br>-GCTA (Genome-wide Complex Trait Analysis, 1.93.2beta)<br>- Miscellaneous: R v3.6.3, R v4.0.1<br>The script for generating the phenotypes used in the EWAS is available via GitHub [https://github.com/genepi-freiburg/ckdgen-pheno-ewas].

For manuscripts utilizing custom algorithms or software that are central to the research but not yet described in published literature, software must be made available to editors and reviewers. We strongly encourage code deposition in a community repository (e.g. GitHub). See the Nature Portfolio guidelines for submitting code & software for further information.

Data

Policy information about availability of data

All manuscripts must include a data availability statement. This statement should provide the following information, where applicable:

- Accession codes, unique identifiers, or web links for publicly available datasets
- A description of any restrictions on data availability
- For clinical datasets or third party data, please ensure that the statement adheres to our policy

The individual participant data included in this project are generally not publically available due to data protection laws, but can be applied from the individual studies on reasonable request. The summary statistics from the meta-analysis are available in the CKDGen Consortium website (https://ckdgen.imbi.uni-freiburg.de).

Field-specific reporting

Please select the one below that is the best fit for your research. If you are not sure, read the appropriate sections before making your selection.

- [x] Life sciences
- [ ] Behavioural & social sciences
- [ ] Ecological, evolutionary & environmental sciences

For a reference copy of the document with all sections, see nature.com/documents/nr-reporting-summary-flat.pdf

Life sciences study design

All studies must disclose on these points even when the disclosure is negative.

Sample size	Discovery project using maximum sample size available when starting the project. Therefore, no sample-size calculation was performed.
Data exclusions	We excluded studies from an analysis if the sample size for EWAS was not greater than 50, to ensure a reasonable sample size for the statistical association models. DNA methylation probes that are only in the HM450K or EPIC arrays were excluded.
Replication	Replication of significant EWAS results was performed in independent samples of cohorts that were available at time when that replication stage was initiated, and not included in the discovery stage. All results of CpG sites (replicated or not) of the replication stage are provided in the Supplementary Tables.
Randomization	Not applicable because this is an observational study.
Blinding	Not applicable because this is an observational study.

Reporting for specific materials, systems and methods

We require information from authors about some types of materials, experimental systems and methods used in many studies. Here, indicate whether each material, system or method listed is relevant to your study. If you are not sure if a list item applies to your research, read the appropriate section before selecting a response.

Materials & experimental systems

n/a	Involved in the study	
x		Antibodies
x		Eukaryotic cell lines
x		Palaeontology and archaeology
	x	Animals and other organisms
	x	Human research participants
x		Clinical data
x		Dual use research of concern

Methods

n/a	Involved in the study	
x		ChIP-seq
x		Flow cytometry
x		MRI-based neuroimaging

Animals and other organisms

Policy information about studies involving animals; ARRIVE guidelines recommended for reporting animal research

Laboratory animals	ABCG2 Q141K (ABCG2 Q140K) was knocked in to the endogenous Abcg2 locus using CRISPR/ Cas9 on a C57BL/6J background. Species: C57BL/6J, sex: male
Wild animals	None
Field-collected samples	None

Ethics oversight | University of Maryland School of Medicine Institutional Animal Care and Use Committee.

Note that full information on the approval of the study protocol must also be provided in the manuscript.

Human research participants

Policy information about studies involving human research participants

Population characteristics	Details of each cohort are provided in Supplementary Table 1
Recruitment	Participants were recruited prior and independent to this projects, and predominantly population based. Details incl. references are provided in the Supplementary Table 1
Ethics oversight	Each study was approved by the respective ethics committee, and all participants provided written informed consent.

Note that full information on the approval of the study protocol must also be provided in the manuscript.

March 2021
